# Accounting for Capacity Constraints in Economic Evaluations of Precision Medicine: A Systematic Review

**DOI:** 10.1007/s40273-019-00801-9

**Published:** 2019-05-13

**Authors:** Stuart J. Wright, William G. Newman, Katherine Payne

**Affiliations:** 1 Manchester Centre for Health Economics, Division of Population Health, Health Services Research and Primary Care, The University of Manchester, Oxford Road, Manchester, M13 9PL UK; 2 Manchester Centre for Genomic Medicine, Division of Evolution and Genomic Sciences, The University of Manchester, Manchester, UK; 3 North West Genomic Laboratory Hub, Manchester Centre for Genomic Medicine, Manchester University NHS Foundation Trust, Manchester, UK

## Abstract

**Background and Objective:**

Precision (stratified or personalised) medicine is underpinned by the premise that it is feasible to identify known heterogeneity using a specific test or algorithm in patient populations and to use this information to guide patient care to improve health and well-being. This study aimed to understand if, and how, previous economic evaluations of precision medicine had taken account of the impact of capacity constraints.

**Methods:**

A meta-review was conducted of published systematic reviews of economic evaluations of precision medicine (test–treat interventions) and individual studies included in these reviews. Due to the volume of studies identified, a sample of papers published from 2007 to 2015 was collated. A narrative analysis identified whether potential capacity constraints were discussed qualitatively in the studies and, if relevant, which quantitative methods were used to account for capacity constraints.

**Results:**

A total of 45 systematic reviews of economic evaluations of precision medicine were identified, from which 222 studies focusing on test–treat interventions, published between 2007 and 2015, were extracted. Of these studies, 33 (15%) qualitatively discussed the potential impact of capacity constraints, including budget constraints; quality of tests and the testing process; ease of use of tests in clinical practice; and decision uncertainty. Quantitative methods (nine studies) to account for capacity constraints included static methods such as capturing inefficiencies in trials or models and sensitivity analysis around model parameters; and dynamic methods, which allow the impact of capacity constraints on cost effectiveness to change over time.

**Conclusions:**

Understanding the cost effectiveness of precision medicine is necessary, but not sufficient, evidence for its successful implementation. There are currently few examples of evaluations that have quantified the impact of capacity constraints, which suggests an area of focus for future research.

**Electronic supplementary material:**

The online version of this article (10.1007/s40273-019-00801-9) contains supplementary material, which is available to authorized users.

## Key Points for Decision Makers

**Table:** 

Examples of precision medicine are complex interventions and limited health system capacity may impede their adoption into clinical practice.
Capacity constraints may have an impact on the cost effectiveness of examples of precision medicines and should be included in economic evaluations of such interventions.
Evidence as to the value of removing capacity constraints over time in terms of improving the cost effectiveness of examples of precision medicine may be also be useful to decision makers in guiding strategies to improve implementation.

## Introduction

Precision (stratified or personalised) medicine is underpinned by the premise that it is feasible to identify known heterogeneity using a specific test or algorithm in patient populations to guide patient care to improve health and well-being [1]. There is no unified definition of precision (stratified or personalised) medicine, but in practice to date it refers to using some test–treat combination to target an intervention [1, 2]. A variety of mechanisms are undergoing development to identify such heterogeneity in the outcomes of interventions and progression of disease in populations of patients informed by genomic, proteomic, transcriptomic and metabolomic strategies [2]. The ability to determine which patients might be more likely to benefit from a treatment, avoid adverse effects or experience more severe disease has driven the theoretical arguments that precision medicine is a cost-effective use of healthcare resources [3, 4].

Determining the incremental cost effectiveness of the strategies to deliver precision medicine (hereafter shortened to precision medicine) is important because diverting funding to such newer interventions will involve the reallocation of resources from other areas of medicine. The reallocation of funding may affect the health outcomes for relevant populations of patients, representing the opportunity cost of the new intervention. Economic evaluations provide a structured framework to provide evidence supporting whether the introduction of precision medicine is an effective use of healthcare resources.

There is some economic evidence supporting exemplars of precision medicine [5]. However, even with such economic evidence, the introduction of precision medicine into health systems has been slower than anticipated, potentially due to the volume of eligible patients. There is emerging anecdotal, and some empirical, evidence of factors limiting the uptake of precision medicine. In 2015, the charity Cancer Research UK (CRUK) published a report highlighting the significant delays in providing genetic mutation testing to patients [6] and estimated that in the previous year approximately 3500 patients may have missed out on receiving a medicine that may have improved their quality and length of life because of the absence of relevant mutation testing. Questions about the ability of the UK National Health Service (NHS) to implement one of the case study examples of precision medicine, *EGFR* mutation testing and gefitinib, a specific epidermal growth factor receptor (EGFR) inhibitor licenced for patients with *EGFR* mutation-positive non-small cell lung cancer, without “substantial investment in time and resources” have been raised. It was believed that the NHS did have capacity to introduce such testing in the short timeframe [7]. However, this proved not to be the case, with the CRUK report suggesting that only 52% of patients received *EGFR* mutation testing even 4 years after the approval of gefitinib by the National Institute for health and Care Excellence (NICE) [6]. This case study introduces the potential for capacity constraints to be a key barrier to the introduction of precision medicine, even when these have been shown to be cost effective in clinical practice.

No consensus definition of what constitutes a capacity constraint in the context of healthcare interventions exists in the literature. The *Oxford English Dictionary* (OED) defines capacity as “the amount that something can produce” [8]. This definition implies that it is necessary to be clear what is being produced. The capacity of a healthcare system could therefore be defined as its ability to produce some defined output. In keeping with the extra-welfarist normative underpinning, assumed by decision-making bodies such as NICE, the relevant output of a healthcare system has been defined as ‘health status’ measured using the quality-adjusted life-year (QALY). The definition of a constraint is “something that controls what you do by keeping you within particular limits” [9]. Combining the definitions of capacity and constraint can be used to propose a working definition of a capacity constraint in a healthcare system which has the goal of maximising health status: ‘Any factor which impedes or limits the amount of health status produced for a population of patients receiving specified interventions, or policies, provided by the healthcare system’.

The introduction of any healthcare intervention may be impeded by capacity constraints in a healthcare system, which may be particularly extensive and significant for examples of precision medicine due to their nature as complex interventions involving both a test and treatment element [10]. There is some qualitative evidence describing the type of capacity constraints directly relevant to the uptake of precision medicine into practice, including a lack of laboratories providing tests, poor logistics resulting in slow test turnaround, a lack of training for clinicians, and insufficient funding for testing or treatments [11–13]. The impact of such capacity constraints on the incremental cost effectiveness of precision medicine has not been well-described. This study aimed to identify a sample of published economic evaluations of precision medicine and describe if, and how, these economic evaluations had qualitatively discussed and quantitatively accounted for capacity constraints in the analysis.

## Methods

This study used a two-stage systematic review conducted and reported in accordance with published guidelines and reporting criteria [14]. A published search of PubMed by Payne and colleagues [5] suggested that there were a substantive number of previously published systematic reviews of economic evaluations of precision medicine and related areas such as personalised medicine, pharmacogenetics and pharmacogenomics. Therefore, a de novo systematic review that aimed to identify all previous economic evaluations of precision medicine would overlap significantly with this previous body of work, requiring significant resources in terms of researcher time but unlikely to yield substantively different findings. This study therefore used a strategy to identify and collate published systematic reviews of economic evaluations of precision medicine. From these systematic reviews a sample of economic evaluations of test and treatment-based examples of precision medicine were identified. The identified sample of economic evaluations were published between 2007, when the use of terms related to precision medicine began to occur regularly in the literature [2], and February 2017.

For the purpose of this review precision medicine was defined as an intervention that uses, for example, a test to “identify subgroups of patients with distinct mechanisms of disease, or particular responses to treatments” [1]. Related areas included were therefore as follows: precision medicine; stratified medicine; individualised medicine; genetic medicine; genomic medicine; personalised medicine; and targeted medicine [15]. This systematic collation of systematic reviews published up to February 2017 was then used to identify an exemplar sample of individual economic evaluations and this sample was used to identify if, and how, capacity constraints had been included in the published analyses. The review involved two stages. Stage one involved the systematic collation of a sample of systematic reviews of economic evaluations of precision medicine. Stage two involved creating a list of relevant individual economic evaluations of precision medicine.

### Stage One

Table 1 summarises the inclusion criteria used to guide the relevance of published systematic reviews of economic evaluations of precision medicine. To be classified as a systematic review, the published study must have used a systematic approach to search databases of published literature with the aim of identifying all studies that addressed a specified research question.

**Table 1:** Inclusion criteria for systematic reviews of economic evaluations of precision medicine

Aspect of study	Inclusion criteria
Population	Any relevant group of patients
Intervention	A stratifying test, algorithm or test–treatment combination
Comparator	Current practice
Outcomes	Costs and consequences relevant to a full economic evaluation (cost–utility analysis; cost-effectiveness analysis; cost–benefit analysis)
Study type	Systematic review
Availability	English: full text

#### Search Strategy

The MEDLINE (inception year: 1946) and EMBASE (inception year: 1980) databases were searched from database inception to February 2017, using an electronic search strategy, to identify all systematic reviews of economic evaluations of precision medicine as defined by this review. The electronic search strategy for this systematic review was based on a published economic evaluation search filter developed by the University Of York-based Centre for Reviews and Dissemination (CRD) [16] and combined with terms relevant to precision medicine and a systematic review search filter, which were informed by a published strategy [5] (see Electronic Supplementary Material (ESM) Appendix 1). In addition, a selection of lead or senior (last) authors of published systematic reviews of economic evaluations of precision medicine were contacted by e-mail to determine whether they knew of other published systematic reviews not identified in the initial search relevant to the selected time period.

#### Selection Process

The abstracts identified in the electronic literature search were screened for relevance and inclusion in the review by a team of three reviewers at the Manchester Centre for Health Economics (SJW, Hunter Moore and Sean Gavan). Each abstract was screened by two of the three reviewers. Disagreements on whether a study should be included were resolved by a fourth researcher (Niall Davison).

#### Data Extraction and Analysis

The number of systematic reviews of economics evaluations of precision medicine was recorded and their key details summarised using a table (ESM Appendix 2). The identified systematic reviews were listed and then categorised into one of four categories depending on their stated focus of the systemic review of economic evaluations: (1) test and treat interventions across disease areas; (2) test only interventions across disease areas; (3) test and treat interventions within a given disease area; and (4) test only interventions within a given disease area. Details regarding the number of primary economic evaluations cited in each review were also recorded. These findings were then presented using a narrative summary.

### Stage Two

Table 2 summarises the inclusion and exclusion criteria for the individual studies to define a sample of economic evaluations of test and treatment-based precision medicine. Studies that focused on interventions which provide diagnostic information that has no impact on treatment were excluded from this study. A second restriction applied because of the volume of individual economic evaluations limited the identified studies to those published in the 10 years prior to the date of the search.

**Table 2:** Inclusion criteria for primary economic evaluations of precision medicine

Aspect of study	Inclusion criteria
Population	Any relevant group of patients
Intervention	A stratifying test or algorithm used to subsequently guide a specified treatment or type of treatments
Comparator	Current practice
Outcomes	Costs and consequences relevant to a full economic evaluation (cost–utility analysis; cost-effectiveness analysis; cost–benefit analysis)
Study type	Primary economic evaluations (prospective or model-based)
Availability	English: full text
Timeframe	Published during or after 2007 and up to February 2017

#### Search Strategy

A list of individual economic evaluations of precision medicine were identified from the reference lists of the systematic reviews identified in stage one. Grey literature studies that had been identified in the systematic reviews were retained if they met the inclusion criteria for individual studies. This involved a manual search facilitated using a database created in Microsoft Excel^®^ 2010 [17].

#### Selection Process

To determine the relevance of the individual studies and inclusion of primary economic evaluations of test and treatment strategies, the abstracts of the identified studies were imported into the bespoke database in Excel^®^ and then double screened by two reviewers (SJW and Martin Eden, Manchester Centre for Health Economics), with disagreements resolved by a third reviewer (KP).

To identify studies that had discussed capacity issues, a manual keyword search of the PDF for each included study was conducted. To identify relevant keywords, a search of the title and abstracts of relevant theoretical papers was conducted using the Mendeley reference management software [18–33]. The identified terms were capacity, barrier*, constrain*, restrict*, short (for short-run or short-term), implement*, learn*, inefficien*, bottleneck, scale, utilis*, utiliz*.

#### Data Extraction and Analysis

The individual studies were initially collated into one of four categories according to the aim of the parent systematic review (as outlined in the stage one data extraction). It was clear, however, that some systematic reviews had identified some studies as test–treat when they were test-only strategies (and vice versa). Therefore, after review of the titles and abstracts of the individual studies, they were then reclassified into one of three categories: (1) economic evaluations of test and treatment interventions; (2) economic evaluations of test-only interventions; and (3) other studies that did not meet the eligibility criteria for this systematic review. Studies classified into the second and third categories were then excluded from this review.

The total number of papers identified and their characteristics are summarised in ESM Appendix 3. Data were extracted by one reviewer (SJW) using a data extraction table produced in Microsoft Word^®^ [34]. Data extraction fields included author; year; country; intervention and comparator; whether the study mentioned capacity, and a brief extract where this was so; and whether the study attempted to account for capacity constraints, and a brief description of the method to account for capacity constraints. Due to the large size of this study, the data extraction table provided only reported papers which as a minimum criterion discussed capacity issues in a qualitative manner (ESM Appendix 4). Studies that attempt to quantitatively account for capacity constraints were summarised using the Consolidated Health Economic Evaluation Reporting Standards (CHEERS) statement [35]. Themes in the discussion and analysis of capacity issues in the individual evaluations were identified using thematic analysis of the published text in the manuscript. These themes were then discussed in a narrative summary. The narrative summary described the capacity constraints identified in the literature, the extent of the problem of capacity in economic evaluations, and the methods used to deal with these issues in economic evaluations of precision medicine.

## Results

**Fig. 1:**
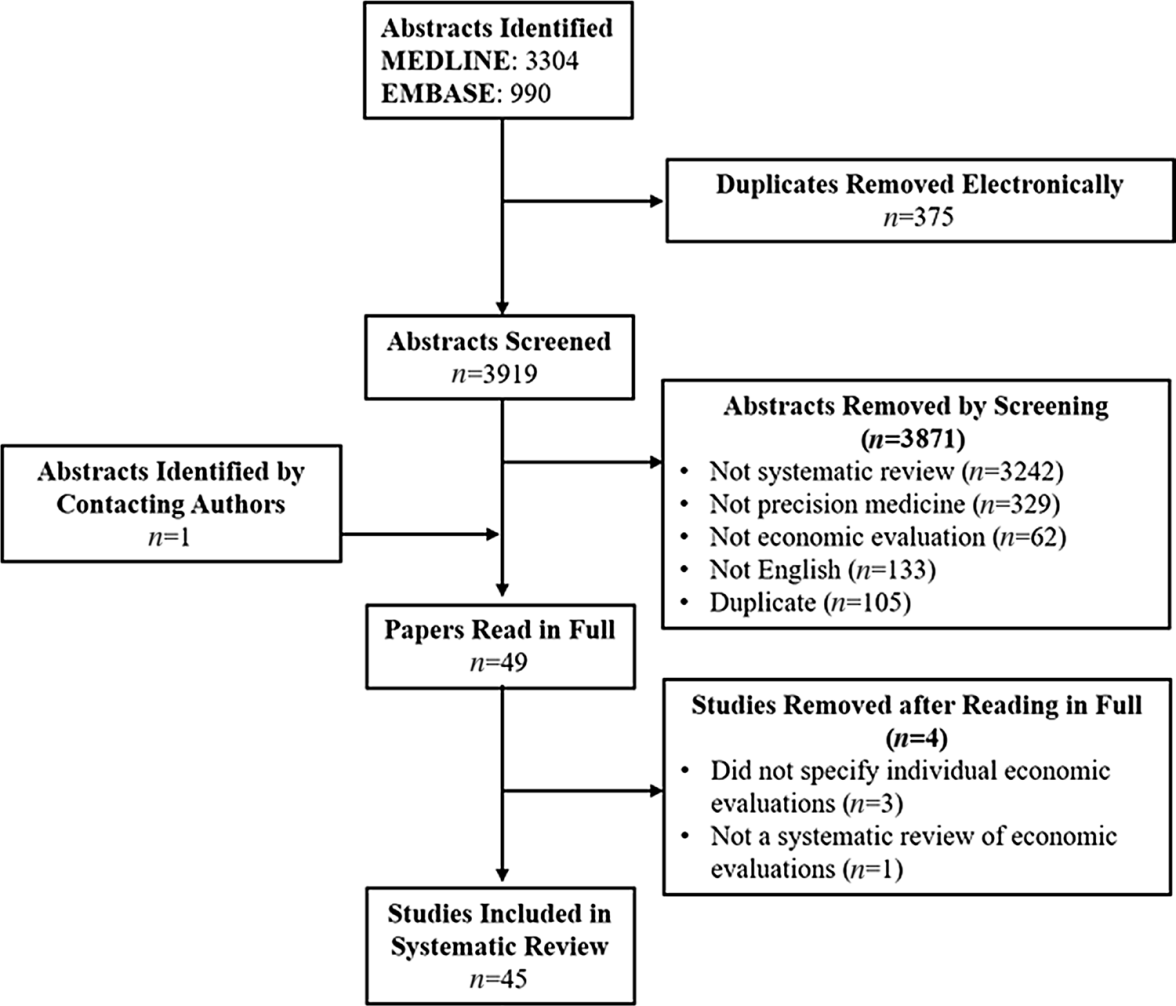
Identification of systematic reviews of economic evaluations of precision medicine

A total of 45 systematic reviews of economic evaluations of precision medicine published up to and including February 2017 were included in this review (ESM Appendix 2). A Preferred Reporting Items for Systematic Reviews and Meta-Analyses (PRISMA) diagram showing the process of identifying the systematic reviews for inclusion in this study is presented in Fig. 1. The initial literature search identified 3304 potentially relevant papers in the MEDLINE database and 990 in the EMBASE database. Microsoft Excel^®^ was used to remove duplicate copies first by abstract (*n* = 283) and secondly by title (*n* = 92), leaving 3919 papers to review. During double screening of the abstracts of the identified papers, 3871 papers were removed: 3242 were not systematic reviews, 329 did not focus on precision medicine, 133 were not in English, 105 were duplicates, and 62 were not economic evaluations. This left 48 systematic reviews of economic evaluations of precision medicine, to which an additional single study was added following direct contact with key authors in the area. Three reviews were subsequently excluded from data extraction because they did not report the citations for the individual studies included in their review, and one study was removed as on closer inspection it became clear it was not a systematic review of economic evaluations.

**Fig. 2:**
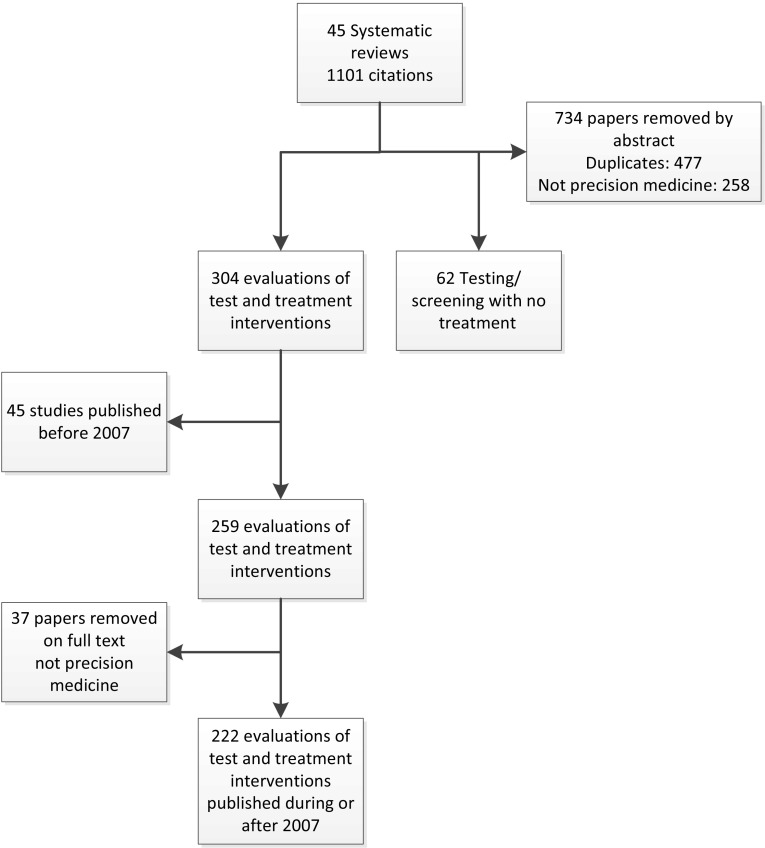
Identification of primary economic evaluations of test and treatment precision medicine

A total of 222 individual economic evaluations of precision medicine involving a test–treat strategy published between 2007 and 2015 were included in this review (see Fig. 2). Extraction of the individual economic evaluations from the identified systematic reviews yielded a list of a total of 1101 studies. From this group 477 papers were duplicates and a further 258 papers were removed as they did not evaluate an intervention relevant to precision medicine as defined in this review. Of the remaining 366 studies, a further 62 studies that reported an economic evaluation of a test-only strategy that did not inform a treatment option were excluded. Restricting the individual economic evaluations of test–treat strategies to those published within the last 10 years (2007–2017) yielded 259 studies. Of these studies a further 37 studies were removed during data extraction as it was clear on reading the full text that the study did not focus on precision medicine. Such studies were economic evaluations of medicines which had, in theory, a precision application but were not being evaluated in this way because the costs and consequences of the ‘test’ element were not included in the evaluation. For example, some studies evaluated the cost effectiveness of erlotinib for all non-small cell lung cancer patients rather than just those with *EGFR* mutations [36, 37].

### Description of Systematic Reviews

A total of 45 systematic reviews of economic evaluations of precision medicine were identified which were published between 2004 and February 2017. There were four categories of reviews of economic evaluations: test and treat interventions across disease areas or technology (*n* = 13); test and treat interventions in a specific disease area of technology (*n* = 17); diagnostic-only interventions across disease areas or technologies (*n* = 9); and diagnostic-only interventions in a specific disease or technology area (*n* = 6). The size of the reviews ranged from zero included papers, for a review of evaluation of genetic diagnosis of aneuploidy in all chromosomes [38], to 140 papers for a review of economic evaluations of targeted and non-targeted therapies for breast cancer [39].

### Description of Individual Economic Evaluations

Of the 222 identified economic evaluations of precision medicine, 159 (71%) used QALYs) as the outcome of interest and 45 (20%) used clinical outcomes. A further 19 (9%) studies used a mixture of outcomes. The majority of the economic evaluations were decision-analytic model based (*n* = 203, 91%), and 20 (9%) were based on primary data from trials. Of the studies using model-based evaluations, 124 (56%) used Markov models, 33 (15%) used decision trees, 17 (8%) used linked decision trees and Markov models, and 16 (7%) were individual-level simulation models. Six studies used other methods ranging from simple quantitative calculations based on literature reviews and meta-analysis to more complex methods such as dynamic life-cycle modelling, a system dynamics-based approach [40]. In ten (4%) studies it was unclear what modelling approach was used.

The individual economic evaluations identified the costs and consequences of precision medicine for 32 distinct conditions or groups of conditions (Table 3). In total, precision medicine targeting cancer comprised 67% (*n* = 151) of the individual studies. Evaluations of precision medicine targeting breast cancer dominated the identified economic evaluations (*n* = 104, 46%). The other common conditions of focus were cardiovascular conditions (*n* = 22, 10%), including acute myocardial infarction, atrial fibrillation and acute coronary syndrome. Collectively, the interventions in these studies were commonly aimed at preventing strokes. Other more commonly considered conditions included lung cancer (*n* = 18, 8%), colorectal cancer (*n* = 15, 7%) and HIV (*n* = 12, 5%).

**Table 3:** Summary of included studies using the Consolidated Health Economic Evaluation Reporting Standards (CHEERs) checklist

Study (year)/country	Intervention and comparator	Study population	Economic evaluation type	Evaluation vehicle (model type if applicable)	Time horizon (discount rate)	Analysis	Approach to quantifying capacity constraints
Delea et al. (2012) [74]/UK	Intervention: lapatinib and capecitabine Comparator: capecitabine monotherapy	Women with *HER2*-positive metastatic breast cancer who have previously received trastuzumab	Cost–utility analysis	Model (partitioned survival analysis)	5 years (3.5%)	Incremental analysis reported: yes PSA: yes Other sensitivity analysis: one-way deterministic	An average drug wastage number was used in the analysis and this was set to zero in sensitivity analysis, reducing total costs. This suggests cost effectiveness of intervention depends on implementation
Delea et al. (2013) [75]/UK	Intervention: lapatinib and letrozole Comparator: trastuzumab and anastrozole or trastuzumab alone or letrozole alone	Women with hormone receptor- and *HER2*-positive metastatic breast cancer	Cost–utility analysis	Model (partitioned survival analysis)	10 years (3.5%)	Incremental analysis reported: yes PSA: yes Other sensitivity analysis: one-way deterministic	An average drug wastage number was used in the analysis and this was set to zero in sensitivity analysis, reducing total costs. This suggests cost effectiveness of intervention depends on implementation
Djalalov et al. (2014) [44]/Canada	Intervention: EML4-ALK fusion testing and first-line crizotinib treatment Comparator: cisplatin and gemcitabine	Patients with advanced ALK-positive NSCLC	Cost–utility analysis	Model (decision tree linked to Markov model)	Lifetime (5%)	Incremental analysis reported: yes PSA: no Other sensitivity analysis: One-way and two-way deterministic	Decision tree includes a branch for whether there is an adequate tissue sample and if not allows for a second biopsy to be taken. It is not clear if these probabilities were varied in sensitivity analysis but the cost of re-biopsy was allowed to vary
Garrison and Veenstra (2009) [76]/USA	Intervention: trastuzumab Comparator: not stated	Women with various stages of breast cancer	Cost–utility analysis	Model (dynamic life-cycle modelling)	10-year product life cycle (3%)	Incremental analysis reported: yes PSA: no Other sensitivity analysis: one-way deterministic	Dynamic cost effectiveness with changing patient population. Implies limited approved indications for drug may inhibit potential cost effectiveness
Lorenzana et al. (2012) [50]/South Africa	Intervention: genotype assay for selection of third-line ART Comparator: all patients receive second-line treatment or all patients receive third-line treatment	ART-naïve cohort of patients with HIV	Cost-effectiveness analysis	Model (discrete event simulation)	Time horizon not stated (3%)	Incremental analysis reported: yes PSA: no Other sensitivity analysis: one-way and multi-way deterministic	Test cost was varied in sensitivity analysis with suggestions that higher test cost could represent cost when investment is accounted for. No impact on cost effectiveness found
McCowan et al. (2013) [73]/UK	Intervention: high adherence (≥ 80%) to tamoxifen Comparator: low adherence (< 80%) to tamoxifen	Women with breast cancer	Cost utility	Model (Markov)	Lifetime (3.5%)	Incremental analysis reported: yes PSA: yes Other sensitivity analysis: one-way deterministic	Evaluation conducted across subgroups of patients with under or over 80% adherence. Low adherence associated with expected loss of 1.12 discounted QALYs and increase of £5970 in medical costs Methods could be extrapolated to compare cost effectiveness of high patient access to treatments
Retèl et al. (2012) [54]/The Netherlands	Intervention: 70-gene MammaPrint assay to guide adjuvant breast cancer treatment Comparator: adjuvant! Online algorithm to guide treatment	Women with breast cancer	Cost utility	Model (linked decision tree and Markov with multiple cohorts and varying parameters)	15 years [4% (costs) and 1.5% (outcomes)]	Incremental analysis reported: yes PSA: no Other sensitivity analysis: none	The researchers modelled the cost effectiveness over time and diffusion of the technology. They include a range of potential scenarios and barriers which affect the diffusion of the technology
Romanus et al. (2015) [57]/USA	Intervention: multiplexed testing for *EGFR* and *ALK* mutations to guide NSCLC treatment Comparator: no testing and treatment with pemetrexed and cisplatin	Patients with NSCLC	Cost utility	Model (discrete event simulation)	2 years (3%)	Incremental analysis reported: yes PSA: no Other sensitivity analysis: one-way deterministic. Threshold analysis for turnaround time for testing	Includes a parameter for turnaround time and inadequate tissue sample leading to re-biopsy as well as proportion of patients tested
Vanderlaan et al. (2011) [72]/USA	Intervention: 21-gene assay to guide adjuvant chemotherapy Comparator: treatment guided by US NCCN guidelines	Women with node-positive, early-stage breast cancer	Cost utility	Model (decision tree)	30 years (3%)	Incremental analysis reported: yes PSA: no Other sensitivity analysis: one-way deterministic	Sensitivity analysis included variations in utilisation rates of testing, although marginal costs were linear so no impact on cost effectiveness

*ART* antiretroviral therapy, *EML4-ALK* echinoderm microtubule associated protein-like 4–anaplastic lymphoma kinase, *NCCN* National Comprehensive Cancer Network, *NSCLC* non-small cell lung cancer, *PSA* probabilistic sensitivity analysis, *QALYs* quality-adjusted life-years

### Inclusion of Capacity Constraints

Of the 222 individual economic evaluations included in this review, 33 mentioned the potential impact of capacity constraints on the costs and consequences of precision medicine in a qualitative sense and nine of these 33 studies went further and attempted to quantify the impact of capacity constraints in the analysis (Table 3). ESM Appendix 4 summarises the 33 included studies. These 33 studies raised key elements related to capacity constraints and how these may impact on the cost effectiveness of precision medicine. These capacity constraints were grouped into four themes: budget constraints; quality of the testing process; ease of test use in clinical practice; and the need for economic evidence to reduce decision uncertainty.

#### Budget Constraints

A key concern relevant to capacity constraints was the total impact of precision medicine on healthcare budgets (budget impact). Budget impact was mentioned in terms of the specific treatment or associated testing and how this may potentially inhibit the uptake and use of these interventions in clinical practice. Three studies focused on the budget impact in broad terms [41–43], but a further ten studies specified the discussion about the financial impact of testing or treatment [42, 44–52].

Kondo et al. [47] stressed the need for concern regarding the financial impact of implementing 12,000 new 21-gene assays for breast cancer per year. The considerably larger eligible patient populations who could benefit from interventions such as warfarin dosing tests were suggested to pose more substantial problems in terms of financial impact. Patrick et al. [53] identified that up to 10 million people could benefit from tests costing between US$400 and US$550 per test.

When evaluating the implementation of the more advanced fluorescent in situ hybridisation (FISH) testing for reflex testing human EGFR 2 (*HER2*) amplifications in breast cancer, Garrison et al. [45] stated that it was unclear whether payers would finance the more rigorous test over the commonly used immunohistochemistry test (IHC). Retèl et al. [54] assessed that there was a 75% chance that a 70-gene signature test for breast cancer would not be immediately reimbursed by payers, thereby limiting the availability of testing to patients.

The introduction of genetic testing for breast cancer was seen by the Medical Advisory Secretariat of the Ministry of Health and Long-Term Care, in Ontario, Canada, as an action that could either release health system resources by reducing the number of women receiving unnecessary chemotherapy, or place an additional burden through additional test costs [51]. This report highlighted potential economic incentives that may impact the level of usage of testing, including pharmaceutical companies’ desire to maintain profits in providing the drug to wider population and payers’ desire to reduce drug expenditure.

The cost of specific treatments was also commonly highlighted as a potential barrier to their full implementation. Ito et al. [46] suggested that the out-of-pocket costs of accessing aromatase inhibitors for breast cancer may cause under-utilisation of the potentially health-improving treatment in the USA. It was determined that improving coverage of these drugs by Medicare, a government health insurance scheme, would improve health outcomes while reducing healthcare resource use.

Lidgren et al. [49] suggested that individual clinicians may decide not to prescribe treatments due their high cost. The authors also suggested that if different healthcare clinics have different financial budgets then provision of trastuzumab for breast cancer may be variable and this could lead to inequitable access to treatment.

#### Quality of Tests and Testing Processes

A commonly mentioned issue linked to capacity constraints and the cost-effective use of precision medicine was the quality of the tests and testing process to determine the appropriateness of treatment. Suboptimal testing may result from factors including a limited supply of trained test providers, such as pathologists and geneticists, and testing facilities, or unclear reporting of test results to prescribing clinicians. The reduced test quality and potential volume of available tests may both serve to reduce the potential health that could be produced by a new precision medicine. In a health technology appraisal report, Collinson et al. [55] outlined the criteria for an effective cardiac biomarker: “A marker suitable for routine clinical use must be measurable in the routine clinical laboratory without special handling conditions”. These authors stated that a biomarker must be measurable with “precision and accuracy, the analysis must be simple and have a rapid turnaround time” [55]. The biomarker test should also ideally be able to be implemented using existing laboratory resources. Eight studies discussed how current testing methods may fail to exhibit such characteristics [44, 45, 50, 55–59].

Garrison et al. [45] suggested that it may not be possible to fully implement reflex FISH testing for *HER2* mutations in breast cancer given the need for additional laboratory resources and expertise. These authors explicitly stated a need for “improvements in capacity and investment in equipment, which may be costly” [45]. The Ontario Medical Advisory Secretariat suggested similar barriers when evaluating FISH testing for non-small cell lung cancer, in particular a lack of expertise in the technique [56]. A similar study by Djalalov et al. [44], based in Canada, highlighted issues with the complexity of testing and the need for sufficient tissue alongside the relatively small number of patients with relevant mutations in a significantly larger population.

Romanus et al. [57], in a US-based study, identified that *EGFR* testing for non-small cell lung cancer was only available for 5.7% of the patients for whom guidelines specify testing should be available. These authors went on to suggest that long turnaround times for the tests used to target the medicine could push the balance of cost effectiveness in favour of generic chemotherapy for all patients. Lala et al. [58] reported that shorter turnaround times for testing were also seen as a factor in making the implementation of point-of-care biomarker testing for adverse cardiovascular events feasible. In a survey conducted as input into the analysis of an economic evaluation, Barone et al. [59], an Italian research team, found that 75% of a sample of oncologists, pathologists, molecular biologists and surgeons believed a delay in receiving *KRAS* mutation testing result for colorectal cancer patients would affect their treatment choice and that 25% experienced such delays in actual practice.

#### Ease of Test Use in Clinical Practice

To effectively introduce a new test into clinical practice, the relevant health professionals must be aware of the test and have sufficient training to offer it and interpret and appropriately use the results in their decision-making to guide clinical practice. Insufficient human capital can therefore act as a capacity constraint in moving precision medicine into practice. When tests and treatments are assessed in trials and economic evaluation the assumption is that they are used in an optimal, or near optimal, way, and with full adherence to their recommended use by clinicians. However, clinical practice is complex and the way in which a test fits into the current care pathway is not always clear. Nine studies highlighted issues with the transition of tests from research to clinical settings [41, 42, 54, 55, 60–64]. For example, Breijer et al. [61] developed and evaluated two multivariable models to produce an algorithm to predict the risk of endometrial cancer based on patients’ characteristics. While this analysis suggested a reduction in the cost of diagnosing the cancer, these authors proposed that due to the minimal expected value per patient, the algorithm would have to be made very easy to use by clinicians for it to be implemented in clinical practice.

The potential reluctance of clinicians to change their prescribing behaviour was shown in a UK study of thiopurine-methyl transferase (TMPT) genotyping to inform the use of azathioprine and the appropriate dosing strategy [62]. Even though the test could be used to better predict which patients would experience profound neutropenia as a reaction from azathioprine, clinicians did not use the test to change the dosing of azathioprine. The authors hypothesised that this is because the clinicians chose to remain conservative with azathioprine prescription due to the other potential adverse reactions that the medicine could cause.

Retèl et al. [54] predicted that the use of a 70-gene signature in breast cancer patients would be delayed by “hesitant adopters” who would not use the results of the assay in decision-making. However, these authors included a scenario in their analysis that assumed an increase in the ease of use of the 70-gene signature would subsequently increase the use of test results in clinical decision-making, resulting in improved cost effectiveness of the intervention [54].

Two studies suggested that the willingness of clinicians to use new tests and their ability to effectively use results in decision-making may evolve over time as a result of a learning process [63, 65]. Klang et al. [63] found that clinicians using the Oncotype DX panel did not register treatment decisions for the first 55 of 368 patients as the clinicians were “learning about the technology and how to interpret the results”. The authors of a US study investigating the use of a multi-gene assay in breast cancer explicitly highlighted that previous analyses of Oncotype DX were based on the assumption that the test would be used as dictated by guidelines [65]. They further stated they believe policy makers were interested in “learning how the assay affected outcomes and costs compared with actual practice and after some period of experience with the assay”. Both of these studies indicated that a lower utilisation of testing by health professionals may impact on the incremental cost effectiveness of precision medicine in clinical practice.

#### Decision Uncertainty

When a new precision medicine is introduced, decision makers, such as individual clinicians, local hospital trusts or national health technology assessment (HTA) agencies, must decide under conditions of uncertainty whether to provide the intervention to patients (implementation). There is a significant cost associated with making the wrong decision related to implementation, which could include reduced patient outcomes at an individual level or societal loss of health from funding cost-ineffective interventions. While commonly used methods such as deterministic and probabilistic sensitivity analysis allow for uncertainty in model parameters to be visualised, a wide range of other uncertainties such as methodological and structural uncertainty may be present in the evaluation of precision medicine [66, 67]. Decision uncertainty around implementation can therefore act as a significant capacity constraint to the introduction of new examples of precision medicine.

As a rationale for conducting economic evaluations, many studies highlighted that evidence of the cost effectiveness of an intervention was a requirement for implementation in a clinical setting [68, 69]. As such, a lack of such evidence on incremental cost effectiveness could inhibit the use of precision medicine in clinical practice. In Canada, the Ontario Medical Advisory Secretariat suggested that evidence on the net resource implications of *HER2* testing for breast cancer was required before the intervention could be adopted into “dynamic health systems” [51].

The published evidence also recognised that economic evaluations are conducted at a certain stage in the development of the intervention to deliver precision medicine and that the use and cost effectiveness may change in subsequent clinical practice. Retèl et al. [54, 70] highlighted that their results were based on the assumption of full implementation and that an evaluation using real-life scenarios was in progress at the time of publishing this study.

In theory, the availability of economic evidence can help to fuel increased implementation of precision medicine but, in addition, increased implementation can itself provide economic benefits. Rubinstein et al. [71] identified that if decision makers relied on a passive diffusion of *BRCA* 1/2 genetic testing into the healthcare system, then potentially the economies of scale which could have been generated from a more managed implementation strategy could be forgone. These authors also suggested that increased implementation of an alternative intervention magnetic resonance imaging in breast cancer may create additional costs and benefits if the imaging becomes a part of the cancer management pathway alongside *BRCA* 1/2 genetic testing. This synergistic effect is known as an economy of scope, whereby the same resource can be used to produce or improve multiple services to provide better outcomes.

### Quantifying the Impact of Capacity Constraints in Economic Evaluations

Nine (see Table 3) of the identified 222 economic evaluations of precision medicine included in this systematic review used, or suggested, techniques that explicitly quantified the impact of capacity constraints [44, 50, 54, 57, 72–76]. All of these studies used decision-analytic models that allowed for sub-perfect implementation of technologies, limiting their potential benefit to society. In other words, for various reasons, fewer than 100% of the eligible patient population were assumed to receive the intervention delivering precision medicine or the intervention was not given in the optimal way, resulting in higher costs or lower benefits than were potentially achievable. In some cases this less than perfect implementation was due to capacity issues such as budget constraints [54], regulation barriers [54] and long test turnaround times [57]. In two other instances, the imperfect implementation was associated with low uptake of the test or treatment [72, 73]. While adherence to medications may appear to be a demand-side problem, it is in fact a complex issue and “the attributes of the health-care system and service delivery may also influence adherence” [73]. For example, if clinicians have limited time to spend with patients, the opportunity to provide effective information about the benefits of adherence and approaches to coping with adverse effects may be limited. The methods used to account for capacity constraints can be categorised as static or dynamic and are described in Sects. 3.4.1 and 3.4.2.

#### Static Methods to Account for Capacity Constraints

Static methods refer to methods used to produce a single cost-effectiveness estimate which takes account of imperfect implementation for one cohort of patients. This estimate of the incremental cost effectiveness may differ from the estimated incremental costs and consequences for a perfectly implemented precision medicine. For example, Delea et al. [74, 75] accounted for drug wastage in their model-based economic evaluation of lapatinib and letrozole for women with *HER2*-positive breast cancer. This analysis identified that reducing wastage of trastuzumab from 15 to 0% resulted in lapatinib and letrozole being cost effective at £22,895 per QALY gained when compared with trastuzumab, which had been a cost-saving intervention in the base-case analysis.

Romanus et al. [57] accounted for specific capacity constraints to multiplexed biomarker testing for non-small cell lung cancer in a sensitivity analysis. When turnaround time increased by a factor of 1.5, the most cost-effective approach changed from “test and treat” to “empiric therapy”, in which patients began treatment with a general chemotherapy agent while waiting for test results. Reducing the proportion of patients being tested from 100% to 5.7% did not impact the rank ordering of the incremental cost effectiveness of the interventions. Likewise, in a study by Vanderlaan and colleagues [72] assumptions about the differences in the uptake of a 21-gene assay for breast cancer did not impact on the incremental cost effectiveness of the intervention. In two further studies, assumptions about the cost of the test or re-biopsy did not affect the incremental cost effectiveness of a genotype assay for drug selection in patients with HIV [50] or EML4 (echinoderm microtubule associated protein-like 4)–ALK (anaplastic lymphoma kinase) testing for patients with non-small cell lung cancer [44].

McCowan et al. [73] found that adherence to tamoxifen in women with breast cancer significantly impacted the incremental cost effectiveness. On average, patients with an adherence of less than 80% to the treatment were expected to experience 1.12 fewer QALYs than those patients assumed to have over 80% adherence. Furthermore, such patients were expected to experience significantly higher medical costs (£5970, 95% confidence interval 4644–7372).

#### Dynamic Methods to Account for Capacity Constraints

Dynamic methods refer to methods that account for capacity constraints that allow the impact of barriers or constraints to change over time and/or in multiple patient cohorts. As a result, the cost effectiveness of the technology also potentially changes over time. The most comprehensive investigation of capacity constraints and impact on implementation in an economic evaluation using dynamic methods was conducted by Retèl and colleagues [54] who investigated the future potential uptake of a 70-gene signature test in breast cancer. This study used an analytical approach called scenario drafting to identify potential barriers and facilitators to the implementation of the technology. These authors constructed different sets of model parameters to reflect the implications of these scenarios, accounting for their perceived likelihood of occurring in reality. These scenarios comprised potential capacity issues such as a lack of reimbursement of testing, regulation issues, uncertainty in the clinical utility of the test, and a lack of use by clinicians due to the difficulty interpreting the tests. The authors also investigated the potential impact of using the test at different stages of the clinical pathway.

The incremental cost effectiveness of the 70-gene signature test was evaluated at different timepoints and implementation levels for three key scenarios: reducing technical failure rates of the test over time; decreasing non-compliance with discordant test results; and increasing financial reimbursement and clinicians’ uptake of testing. The cost and consequences achieved by testing an additional patient was allowed to vary. As each scenario involved the intervention diffusing into clinical practice at different rates, the costs, health outcomes and cost effectiveness of the intervention differed by year and scenario. A key finding of the study was that the intervention would only be cost effective if use of the test results by clinicians improved over time. At the initial time point, 2005, the intervention was always cost ineffective with an incremental cost-effectiveness ratio (ICER) of €1.9 million. If uptake did not improve, then the ICER only improved to €1.5 million in the best-case scenario. In the scenario where other factors remained equal and uptake improve from 3% in 2005 to 50% in 2010 and 92% in 2010, the ICER reduced from €1.9 million to €26,145 and €11,123, respectively. Failing to overcome the capacity constraints of low financial reimbursement of the test and a lack of uptake by clinicians would therefore mean a potentially cost-effective intervention should not be provided as it would result in a societal loss of health to the population. Removal of the capacity constraints makes the intervention cost effective, but the cost of such capacity investments would also need to be accounted for in an economic evaluation.

## Discussion

Precision medicine is often reported to offer a cost-effective approach for the management of a selection of different diseases by using a stratification mechanism to identify which patients may accrue more benefits in terms of response or avoidance of adverse events. Some economic evidence supporting this premise is provided by the growing number of economic evaluations conducted in the past 10 years. This review identified 45 systematic reviews of economic evaluations of precision medicine, which summarised 367 unique individual economic evaluations. Between 2007 and 2015, some 222 economic evaluations were conducted to identify the costs and consequences of test and treat interventions.

The first necessary condition for the adoption of an example of precision medicine is demonstration of whether it is a cost-effective use of healthcare resources using appropriate methods of economic evaluation to identify incremental costs and consequences. However, capacity constraints in a healthcare system have the potential to impact on the estimated incremental cost effectiveness of examples of precision medicine in practice. Only 33 studies of a sample of 222 identified economic evaluations qualitatively discussed the potential impact of capacity constraints for the introduction of examples of precision medicine. The core capacity constraints outlined in these papers included budget constraints; quality of tests and testing processes; ease of test use in clinical practice; and decision uncertainty. Even when interventions appeared cost effective, it was suggested they can pose significant financial burdens on payers who have to rapidly provide access to testing services and potentially expensive treatments.

Despite the potential for capacity constraints to affect the incremental cost effectiveness of precision medicine, only nine (4%) economic evaluations from the sample of 222 sought to quantify the effects of limited capacity. All of the nine studies that quantified the effects of limited capacity were based on decision-analytic models rather than trials, and a wide range of model types including decision trees, Markov models and discrete event simulation were used. The presence of these commonly used models in this review suggests that it would be possible to incorporate capacity constraints in many economic evaluations.

While the way in which capacity constraints are included in models will depend on the model type, the methods used could be categorised as either a static or dynamic approach. The static methods included real-world cost-effectiveness analysis and sensitivity analysis in decision-analytic models. Static approaches to incorporating capacity constraints only give a representation of how the constraints impact the cost effectiveness of the intervention for a single cohort of patients at a single point in time. Dynamic approaches to quantifying capacity constraints allow for the fact that health system capacity can change over time and this can have a changing impact on the cost effectiveness of the intervention. The example of Retèl et al. [54] focused on how the level of uptake impacts on the cost effectiveness of the intervention. However, the method used could also be applied to capacity constraints. If the ICER is non-linear and depends on the level of implementation, then any factor that impedes implementation can in theory render the intervention cost ineffective. For example, if numerous repeat *EGFR* mutation tests are required due to insufficient samples at the start of implementation, this will raise the cost of testing and reduce patient benefits due to delays in receiving treatment. With greater communication regarding biopsy requirements and learning by clinicians, better samples could be obtained, reducing costs and improving benefits. Therefore, the ICER will be dependent on the extent to which the intervention is being effectively implemented and the impact of testing knowledge as a capacity constraint. This study shows the key interaction between barriers that impede the use of an intervention and the potential for marginal costs and benefits to vary depending on the level of implementation. The result is situations where capacity constraints and other barriers to implementation cause the intervention to become cost ineffective. Implementing the precision medicine at this level will result in healthcare resources being diverted away from other areas where they could be put to better use, lowering the overall health of patients in the healthcare system.

The Retèl et al. [54] study also raised an important consideration in that actions can be taken to overcome capacity constraints and to improve the incremental cost effectiveness of precision medicine. For example, testing guidelines could be introduced or education programmes used to improve the quality of biopsy samples for testing. Such an intervention itself would have a cost but would also provide benefits in improving the benefits for patients and making the intervention more cost effective. The evaluation of such strategies to improve the implementation of interventions is known as value of implementation analysis [27]. This systematic review did not identify any value of implementation analyses investigating the implementation of precision medicine.

The use of dynamic, multi-cohort decision-analytic models and the value of an implementation approach may provide better evidence than static methods to decision makers in allowing them to understand how best to implement new examples of precision medicine in a cost-effective way by investing in improving health system capacity. In order to forecast the potential capacity constraints before the approval of a precision medicine by an HTA body, the use of qualitative methods such as interviews or focus groups may prove useful. For example, Retèl et al. [54] used the Delphi method with a group of clinicians to identify potential barriers to implementing the MammaPrint test.

### Limitations

The economic evaluation of precision medicine is an expanding research area, and due to the size of the literature base, some restrictions to the inclusion of papers in this study were required. The use of a search strategy that identified previous systematic reviews reduced the number of papers for abstract screening. Collating these studies provided a comprehensive set of primary studies and theoretically all papers previously published in this area. However, due to the length of time required to conduct a systematic review, it is possible that economic evaluations of precision medicine have been published in the time since the literature searches of the most recent systematic reviews were conducted. This means there may be a gap in the studies identified between 2015 and 2017. This could cause bias in the results of this study if methods to include capacity constraints in economic evaluations have been recently developed and applied. The authors of this review are not aware of any recently published novel methods for accounting for capacity constraints in economic evaluations of precision medicine.

The focus on studies published in the last 10 years and evaluations that focused on test and treatment strategies could also feasibly have excluded studies that discussed capacity. While studies published before 2007 may have discussed capacity due to the novelty of precision medicine, it is unlikely that early economic evaluations of precision treatments would have incorporated complex methodological adjustments for capacity. Capacity could be a significant issue in the provision of precision treatments which only provide diagnostic information. Such studies were excluded from this review.

This review has taken a broad definition of a capacity constraint as any factor which impedes the full benefits of an intervention from being realised. While this includes factors such as finite budgets and the quality of the testing process, it also includes more abstract concepts such as low usage of tests due to a lack of knowledge of the technology amongst clinicians. Some of the included studies also investigated adherence to medicine and uptake of treatment. While low adherence could be due to capacity constraints in a lack of patient education about the benefits of treatment, it may also be due to underlying patient preferences for the treatment or adverse effect profile. Therefore, while full implementation of a precision medicine will rely on a lack of capacity constraints on the supply side, it could also be impeded by low demand for the treatment by patients. This could be the case if a new treatment had a greater risk of more adverse effects, more severe adverse effects or a different range of adverse effects. There may, therefore, be a limit to the level of implementation that can be achieved by investing in capacity.

While this review has focused on the impact of capacity constraints for the economic evaluation of precision medicine, such constraints in the healthcare system may have a significant impact on the cost effectiveness of interventions in other medical areas. For example, Jahn et al. [28] explored the cost effectiveness of drug-eluting stents in the presence of capacity constraints using a discrete event simulation model. Capacity constraints may also be particularly significant for the cost effectiveness of organ transplants where there is a limited availability of donors [77]. In addition, capacity constraints and their impact may be larger in countries with developing healthcare systems [78].

## Conclusions

The results of this systematic review suggest that a wide variety of capacity constraints could have implications for the cost effectiveness of precision medicine in clinical practice, but the majority of economic evaluations of precision medicine do not account for such constraints. In the studies that did account for limited health system capacity, a variety of methods had been used, with most relying on static comparisons of the cost effectiveness of examples of precision medicine for a single cohort with or without health system capacity constraints.

Studies should account for changing health system capacity, which may have implications for the cost effectiveness of interventions across multiple cohorts in different years. This is because when combined with varying marginal costs and benefits, implementation-limiting capacity constraints can result in interventions that are cost effective at the population level becoming inefficient in the short-run. Health economists should endeavour to forecast potential barriers to implementing precision medicine and to evaluate potential strategies to invest in capacity. A number of studies have reported qualitative investigations of such barriers [11–13] and combining these approaches with dynamic methods for quantifying such barriers may help to provide decision makers with more robust evidence as to how to cost-effectively implement such interventions and take resource and capacity constraints into account.

## Electronic supplementary material

Below is the link to the electronic supplementary material.

Supplementary material 1 (DOCX 17 kb)

Supplementary material 2 (DOCX 32 kb)

Supplementary material 3 (DOCX 37 kb)

Supplementary material 4 (DOCX 33 kb)

## Data Availability

The list and references of identified systematic reviews of precision medicine are available in Electronic Supplementary Material Appendix 2. The references of included economic evaluations of examples of precision medicine are available in Electronic Supplementary Material Appendix 3.
