# Supplementary material for: Accounting for Capacity Constraints in Economic Evaluations of Precision Medicine: A Systematic Review
Source: Pharmacoeconomics. 2019 May 13;37(8):1011–27. doi: 10.1007/s40273-019-00801-9 (PMC6597608; doi:10.1007/s40273-019-00801-9)
Supplement: Supplementary file 1 — Supplementary material 1 (DOCX 17 kb) [file 40273_2019_801_MOESM1_ESM.docx]

PharmacoEconomics. Accounting for Capacity Constraints in Economic Evaluations of Precision Medicine: a Systematic Review. Stuart J Wright, William Newman, Katherine Payne

Correspondence to Stuart J Wright, Manchester Centre for Health Economics, Division of Population Health, Health Services Research & Primary Care, The University of Manchester, Oxford Road, Manchester, M13 9PL, stuart.wright-2@manchester.ac.uk, 01613067970

Supplementary Appendix 1: Systematic Review Search Strategies

| MEDLINE | EMBASE |
| --- | --- |
| CRD MEDLINE Review Terms  1. review.ti,ab.  2. review.pt  3. meta-analysis.ab  4. meta-analysis.pt  5. meta-analysis.ti  6. or/1-5  7. letter.pt  8. comment.pt  9.editorial.pt  10. or/7-9  11. 6 not 10    EED Economic Terms  12. economics/  13. exp "costs and cost analysis"/  14. Economics, Dental/  15. exp economics, hospital/  16. Economics, Medical/  17. Economics, Nursing/  18. Economics, Pharmaceutical/  19. (economic$ or cost or costs or costly or costing or price or prices or pricing or pharmacoeconomic$).ti,ab.  20. (expenditure$ not energy).ti,ab.  21. value for money.ti,ab.  22. budget$.ti,ab.  23. or/12-22  24. ((energy or oxygen) adj cost).ti,ab.  25. (metabolic adj cost).ti,ab.  26. ((energy or oxygen) adj expenditure).ti,ab.  27. or/24-26  28. 23 not 27  29. exp animals/ not humans/  30. 28 not 29  31. bmj.jn.  32. "cochrane database of systematic reviews".jn.  33. health technology assessment winchester England.jn  34. or/31-33  35. 30 not 34  Genetic, Genomc and Precision Medicine Terms  36. ((genetic$ or pharmacogen$ or pharmaco-gen$) adj2 (test$ or tech$ or assessment or evaluation or intervention$ or screen$ or service$)).mp.  37. biomarker.mp.  38. (next adj generation adj sequencing).mp.  39. (high adj throughput adj sequencing).mp.  40. (whole adj2 sequencing).mp.  41. or/36-40  42. (genom$ or precision or personali$ or stratif$ or individuali$ or target$ or P4) adj (medic$ or treatment or therap$)  43. 41 or 42  44. 11 and 35 and 43 | CRD EMBASE Review Terms  1. exp meta analysis/  2. meta-analys$.ti,ab.  3. metaanalys$.ti,ab.  4. meta analys$.ti,ab.  5. review$.ti.  6. overview$.ti.  7. (synthes$ adj3 (literature$ or research$ or studies or data)).ti,ab.  8. pooled analys$.ti,ab.  9. ((data adj2 pool$) and studies).mp.  10. (MEDLINE or medlars or embase or cinahl or scisearch or psychinfo or psycinfo or psychlit or  psyclit).ti,ab.  11. ((hand or manual or database$ or computer$) adj2 search$).ti,ab.  12. ((electronic or bibliographic$) adj2 (database$ or data base$)).ti,ab.  13. ((review$ or overview$) adj10 (systematic$ or methodologic$ or quantitativ$ or research$ or  literature$ or studies or trial$ or effective$)).ab.  14. 1 or 2 or 3 or 4 or 5 or 6 or 7 or 8 or 9 or 10 or 11 or 12 or 13  15. (retrospective$ adj2 review$).ti,ab,sh.  16. (case$ adj2 review$).ti,ab,sh.  17. (record$ adj2 review$).ti,ab,sh.  18. (patient$ adj2 review$).ti,ab,sh.  19. (patient$ adj2 chart$).ti,ab,sh.  20. (peer adj2 review$).ti,ab,sh.  21. (chart$ adj2 review$).ti,ab,sh.  22. (case$ adj2 report$).ti,ab,sh.  23. (rat or rats or mouse or mice or hamster or hamsters or animal or animals or dog or dogs or  cat or cats or bovine or sheep).ti,ab,sh.  24. 15 or 16 or 17 or 18 or 19 or 20 or 21 or 22 or 23  25. 14 not 24  26. editorial.pt.  27. letter.pt.  28. 26 or 27  29. 25 not 28  30. exp animal/  31. exp nonhuman/  32. 30 or 31  33. exp human/  34. 32 not (32 and 33)  35. 29 not 34  EED Economic Terms  36. Health Economics/  37. exp Economic Evaluation/  38. exp Health Care Cost/  39. pharmacoeconomics/  40. 36 or 37 or 38 or 39  41. (econom$ or cost or costs or costly or costing or price or prices or pricing or pharmacoeconomic$).ti,ab.  42. (expenditure$ not energy).ti,ab.  43. (value adj2 money).ti,ab.  44. budget$.ti,ab.  45. 41 or 42 or 43 or 44  46. 40 or 45  47. note.pt.  48. 46 not 47  49. (metabolic adj cost).ti,ab.  50. ((energy or oxygen) adj cost).ti,ab.  51. ((energy or oxygen) adj expenditure).ti,ab.  52. 49 or 50 or 51  53. 48 not 52  54. 0959-8146.is.  55. (1469-493X or 1366-5278).is.  56. 1756-1833.en.  57. or/54-56  58. 53 not 57  59. conference abstract.pt.  60. 58 not 59  Genetic, Genomic and Precision Medicine Terms  61. ((genetic$ or pharmacogen$ or pharmaco-gen$) adj2 (test$ or tech$ or assessment or evaluation or intervention$ or screen$ or service$)).mp.  62. biomarker.mp.  63. (next adj generation adj sequencing).mp.  64. (high adj throughput adj sequencing).mp.  65. (whole adj2 sequencing).mp.  66. or/61-65  67. (genom$ or precision or personali$ or stratif$ or individuali$ or target$ or P4) adj (medic$ or treatment or therap$)  68. 66 or 67  69. 35 and 60 and 68 |
