# Supplementary material for: Accounting for Capacity Constraints in Economic Evaluations of Precision Medicine: A Systematic Review
Source: Pharmacoeconomics. 2019 May 13;37(8):1011–27. doi: 10.1007/s40273-019-00801-9 (PMC6597608; doi:10.1007/s40273-019-00801-9)
Supplement: Supplementary file 2 — Supplementary material 2 (DOCX 32 kb) [file 40273_2019_801_MOESM2_ESM.docx]

PharmacoEconomics. Accounting for Capacity Constraints in Economic Evaluations of Precision Medicine: a Systematic Review. Stuart J Wright, William Newman, Katherine Payne

Correspondence to Stuart J Wright, Manchester Centre for Health Economics, Division of Population Health, Health Services Research & Primary Care, The University of Manchester, Oxford Road, Manchester, M13 9PL, stuart.wright-2@manchester.ac.uk, 01613067970

**Supplementary Appendix 2: Summary of identified systematic reviews of economic evaluations in precision medicine**

| **Author** | **Year** | **Intervention** | **Reported Health Condition** | **Databases Searched** | **Studies included (n)** | **Reference^a^** |
| --- | --- | --- | --- | --- | --- | --- |
| Antoñanzas et al. | 2012 | Genetic tests | Cystic fibrosis; Breast, Colorectal, Ovarian, and Prostate cancer; Hereditary hemochromatosis; Down’s syndrome; Familial hypercholesterolemia; Thrombophilia | PubMed, Euronheed, University of York (HTA), DARE, NHS EED, Scopus | 51 | [1] |
| Assasi  et al. | 2012 | Predictive, diagnostic, or preventive tests, and genetic tests to guide treatment | Hypercholesterolemia; Prenatal chromosome aberration; Lynch syndrome; Breast, Colorectal, and Non-small cell cancers; Hemochromatosis; Fragile X syndrome; Cytomegalovirus | Medline, Embase, Evidence Based Medicines’s HTA database, PubMed, Wiley’s Cochrane Library, HEED, CRDHTA database | 15 | [2] |
| Beaulieu  et al. | 2010 | Pharmacogenomic tests | Inflammatory bowel diseases; HIV; Thromboembolic diseases; Breast, Colorectal, and Non-small cell lung cancer. | PubMed, Web of Science, Embase | 15 | [3] |
| Berm  et al. | 2016 | Pharmacogenetic and pharmacogenomic screening tests | Specific health conditions not reported | PubMed | 38 | [4] |
| Blank  et al. | 2010 | Conventional and targeted therapies | Metastatic breast cancer | Medline, PubMed | 13 | [5] |
| Bongers  et al. | 2012 | Third-generation therapies (docetaxel, gemcitabine, paclitaxel, pemetrexed, vinorelbine) | Advanced non-small cell lung cancer | PubMed, Embase, HEED | 11 | [6] |
| Carlson  et al. | 2005 | Genetic services (a disease that was primarily genetic or involved a genetic test) | Breast and Colorectal Cancer; Aneuploidies; Cystic fibrosis; Thrombophilia; Fetal anomalies; Hemochromatosis; Hypercholesterolemia; Hemoglobinopathy; Metabolism errors; Rheumatoid arthritis/Lupus | PubMed, Proquest. LexisNexis, Expanded Academic Index, Harvard Review of Economic Analyses, PsycINFO, NICE, CCOHTA | 63 | [7] |
| Coate et al. | 2011 | Agents that target EGFR, VGEF, and novel chemotherapy agents | Non-small cell lung cancer | PubMed, Medline, Google Scholar | 14 | [8] |
| D’Andrea  et al. | 2016 | BRCA testing | Breast and ovarian cancer | Medline, Scopus, HEED, EconLit, HTA, NHS EED | 9 | [9] |
| Degeling  et al. | 2017 | Tests or prediction models to stratify patients into subgroups for screening, treatment targeting, or treatment monitoring | Breast, Lung, Colorectal, and Prostate cancer; Cardiovascular disease; HIV; Hepatitis C; Alzheimer’s disease; Atrial fibrillation; Neonatal disease; Rheumatoid arthritis; Depressive disorder, Type 2 diabetes; Acute myeloid leukemia | PubMed | 31 | [10] |
| Djalalov  et al. | 2011 | Genetic tests or services to detect a heritable disease | Inflammatory bowel disease; Breast and Colorectal cancer; Long QT syndrome; Fragile X syndrome; Schizophrenia; Crohn’s disease; Cystic fibrosis; Hypertension; Nephropathy; Hemochromatosis; Learning disability; Smoking cessation; Aneuploidy; Atrial fibrillation | PubMed, Medline, Proquest, LexisNexis, Expanded Academic Index, The Harvard Review of Economic Analyses, PsycINFO, NICE, CADTH | 26 | [11] |
| Doble  et al. | 2014 | Companion diagnostics of targeted oncology therapies | Breast, Colorectal, and Lung cancer | Cochrane, Evidence Based Medicine Reviews, NHS EED, Embase, Medline, PubMed | 30 | [12] |
| Douglas  et al. | 2016 | Population-wide and high-risk genetic screening | Lynch syndrome; Hereditary breast and ovarian cancer; Familial hypercholesterolemia, Romano-Ward long QT syndrome; Brugada syndrome; Hypertrophic cardiomyopathy; Dilated cardiomyopathy; MYH-associated polyposis; Multiple endocrine neoplasia type 2 | PubMed | 32 | [13] |
| Ferrusi  et al. | 2009 | Trastuzumab and HER2 testing | Breast cancer | Biosis, Cochrane, HEED, CRD, Embase, EconLit, Medline, PubMed | 17 | [14] |
| Ferrusi  et al. | 2011 | Trastuzumab and HER2 testing | Breast cancer | Biosis, Cochrane, CRD, Embase, EconLit, Medline, PubMed, HEED | 14 | [15] |
| Frank  et al. | 2013a | Genomic sequencing technologies | Specific health conditions not reported | Deutsches Ärzteblatt, Biosis Previews, Cochrane Database of Systematic Reviews,  DAHTA-Datenbank, Embase, GMS,  GMS Meetings, Social SciSearch, Health Technology  Assessment Database, SciSearch, Krause & Pachernegg  Verlagsdatenbank, Medline, NHS Economic Evaluation  Database, Thieme Verlagsdatenbank, and Thieme  Verlagsdatenbank PrePrint. | 5 | [16] |
| Frank  et al. | 2013b | Certuximab and panitumumab with and without KRAS testing | Colorectal cancer | DIMDI, Medline, Biosis, GMS, DAHTA, Embase | 7 | [17] |
| Gavan  et al. | 2014 | Explicit stratified approach to treatment | Rheumatoid Arthritis | Medline, Embase, Web of Science, NHS EED | 10 | [18] |
| Giacomini et al. | 2003 | Molecular genetic tests | Breast and ovarian cancer; Thromboembolic disease; Endocrine neoplasia; Hemochromatosis; Cystic fibrosis; Fragile X syndrome; Rheumatic fever; Toxin susceptibility | Medline | 14 | [19] |
| Gonzalez  et al. | 2015 | Genetic testing and electrocardiogram testing for diagnosis | Long QT Syndrome | Medline, Embase, CRD | 4 | [20] |
| Griffith  et al. | 2004 | Cancer genetic services | Familial breast, ovarian, and colorectal cancer | British Medicine Journal Archive, BIDS, MEDLINE, HealthPromis, DARE, EED, HTA, Cambridge Scientific Abstracts, Econobase, CINAHL, ASSIA, British Library Catalogue, WorldCat, Resource Discovery Network, Cochrane library | 25 | [21] |
| Hatz  et al. | 2014 | Individualized medicine – “a therapeutic approach tailoring therapy for genetically defined subgroups of patients” | Breast, Colorectal, Lung, and Ovarian cancer; HIV, Hepatitis C; Atrial fibrillation; Thrombosis; Hypercholesterolemia; Hypertrophic cardiomyopathy; Long QT syndrome; Smoking cessation; Acute coronary syndrome; Nephropathies; Depression; Schizophrenia; Periodontal disease; Cystic fibrosis; Acute lymphoblastic leukemia; MYH-associated polyposis; Epilepsy; Pulmonary fibrosis; Kidney failure; Cardiovascular disease; Hypertension | Medline | 84 | [22] |
| Jarrett  et al. | 2006 | Genetic techniques and testing technologies used for the detection or treatment of diseases with known genetic causes or associations | Breast, Ovarian, and Colorectal cancer; Cystic fibrosis; Down’s syndrome; Remaining diseases not reported | PubMed, Embase, Econlit, NHS EED, Office of Health Economics HEED, NHS HTA | 37 | [23] |
| Lange  et al. | 2014a | Targeted oncology therapies | Non-small cell lung cancer | Medline, Embase, SciSearch, Cochrane Central Register of Controlled Trials and Database of Systematic Reviews, DAHTA, Database of Abstracts of Reviews of Effects, HTA, NHS EED, SOMED, AMED, Biosis | 19 | [24] |
| Lange  et al. | 2014b | Targeted oncology therapies | Colorectal cancer | Medline, Embase, SciSearch, Cochrane Central Register of Controlled Trials and Database of Abstracts of Reviews of Effects, DAHTA, HTA, NHS EED, SOMED, AMED, Biosis | 15 | [25] |
| Langer  et al. | 2010 | PET and CT-based strategies with invasive and non-invasive diagnostic strategies | Solitary pulmonary nodule; Malignant melanoma; Nasopharyngeal carcinoma; Ovarian, Colorectal, Head and neck, Breast, and Non-small cell lung cancer | Cochrane Library, DARE, Embase, HTA Database, NHS EED, PubMed, RePEc, Web of Science | 14 | [26] |
| Lansdorp-Vogelaar  et al. | 2011 | Colorectal cancer screening | Colorectal cancer | Medline, Embase, the Cost-Effectiveness Analysis Registry, British National Health Service Economic Evaluation Database, Lists of technology assessments of the Centers for Medicare and Medicaid Services | 32 | [27] |
| Lee et al. | 2015 | Genetic diagnosis for aneuploidy in all 24 chromosomes (PGD-A) | Aneuploidy in all 24 chromosomes (PGD-A) | Medline, Embase, Scopus, Cochrane Library, NHS EED, EconLit | 0 | [28] |
| Lieberthal | 2013 | Genomic testing | Breast cancer | PubMed, Google Scholar | 9 | [29] |
| Meoni | 2013 | Genetic testing to guide treatment | Infectious diseases | PubMed, CRD | 14 | [30] |
| Nerich  et al. | 2016 | Targeted and non-targeted drug therapies | Breast cancer | Medline, Embase, PubMed, Cochrane Library | 140 | [31] |
| Oosterhoff et al. | 2016 | Biomarkers as a diagnostic (for diagnosis, staging, and as a companion diagnostic) | Colorectal, Lung, Renal, Thyroid, Breast, Endometrial, and Intra-abdominal cancer; Cardiovascular disease; Respiratory disease; Diabetes; Circulatory disease | PubMed, NHS EED | 33 | [32] |
| Parkinson  et al. | 2014 | Trastuzumab | HER2 positive metastatic breast cancer | Medline, Embase, Cochrane Database, EED and HTA | 12 | [33] |
| Payne  et al. | 2009 | TPMT testing for dosing of azathioprine | Crohn’s disease; Rheumatoid arthritis; Systemic lupus erythematous; Inflammatory bowel disease; Acute lymphoblastic leukemia | Medline, Embase, PsychInfo, HAPI, CINAHL | 6 | [34] |
| Philips  et al. | 2004 | Pharmacogenomic interventions | Deep vein thrombosis, Cancer, Viral infections | PubMed  (Medline) | 11 | [35] |
| Plöthner et al. | 2016 | Targeted therapies using pharmacogenomic and pharmacogenetic testing | Epilepsy; Neuropathic pain; HIV; Inflammatory bowel disease; Rheumatoid arthritis; Systemic lupus erythematosus; Acute lymphoblastic leukemia; Breast, Colorectal, and Non-small cell lung cancer | German Institute for Medical  Documentation and Information meta-database | 27 | [36] |
| Plumpton  et al. | 2016 | Pharmacogenetic testing to prevent adverse drug reactions | Acute coronary syndrome; Atrial fibrillation; Acute lymphoblastic leukemia; Crohn’s disease; Cardiovascular disease; Deep vein thrombosis; Inflammatory bowel disease; Major depressive disorder; Myocardial infarction; Colorectal cancer; Peripheral arterial disease; Pulmonary embolus; Rheumatoid arthritis; Stevens-Johnson syndrome; Systemic lupus erythematosus; Ulcerative colitis | Embase, Medline, NHS EED | 47 | [37] |
| Poonawalla et al. | 2015 | Chemotherapeutic agents and targeted biologics | Ovarian cancer | Medline, PubMed, Embase | 28 | [38] |
| Rogowski  et al. | 2006 | Genetic screening | Cystic fibrosis; Diabetes; Hereditary Breast and Ovarian cancer; Retinoblastoma; Familial hypercholesterolaemia; Hereditary haemochromatosis; Hereditary nonpolyposis colorectal carcinoma; Familial adenomatous polyposis colorectal cancer | PubMed, Biosys, Cochrane, DAHTA, Embase, IHTA, Medline, NHS-HTA-DARE, NHS-CRD-HTA, NHS-EED, SOMED | 21 | [39] |
| Smieliauskas et al. | 2014 | Targeted oral anti-cancer medications | Breast cancer; Chronic myeloid leukemia; Gastrointestinal stromal tumor; Hepatocellular carcinoma; Non-small cell lung cancer; Renal cell cancer; Pancreatic cancer | PubMed, Cochrane Library, NIHR HTA | 41 | [40] |
| Stevanovic et al. | 2012 | Risk prediction models | Cardiovascular disease | PubMed, NHS EED | 12 | [41] |
| Vegter  et al. | 2008 | Pharmacogenetic and pharmacogenomic screening programmes | Specific health conditions not reported | PubMed, Embase, Web of Science | 20 | [42] |
| Vegter  at al. | 2010 | Pharmacogenetic and pharmacogenomic screening programmes | Thromboembolism; Atrial fibrillation; Dermatologic conditions; Rheumatologic conditions; Inflammatory bowel disease; Acute lymphoblastic leukemia; Hypercholesterolemia; Nephropathy; Chronic kidney disease; Schizophrenia; Breast, Colorectal, and Lung cancer; HIV; Rheumatoid arthritis; Hypertension; Cystic fibrosis; Thromboembolism; Major depressive disorder; Hepatitis C | PubMed | 42 | [43] |
| Verhoef  et al. | 2010 | Pharmacogenetic-guided dosing of coumarin therapy | Atrial fibrillation, patients starting warfarin | PubMed, Embase, NHS EED, Web of Science | 9 | [44] |
| Wong  et al. | 2010 | Pharmacogenomics | Breast, Colon, and Lung cancer; Rheumatologic conditions; Gastrointestinal diseases; Thromboembolic conditions; HIV; Nicotine addiction; Nephropathy; Psychiatric conditions | PubMed, NICE, Tufts CEA registry, CADTH | 34 | [45] |

References

1. Añtonanzas, F., Rodriguez-Ibeas, R., Hutter, M. F., Lorente, R., Juarez, C., & Pinillos, M. Genetic testing in the European Union: does economic evaluation matter? *The European Journal of Health Economics* **13(5),** 651–661 (2012)
2. Assasi, N., Schwartz, L., Tarride, J., Goeree, R., & Xie, F. Economic Evaluations Conducted for Assessment of Genetic Testing Technologies: A Systematic Review. *Genetic Testing and Molecular Biomarker* **16(11)**, 1322–1335 (2012)
3. Beaulieu, M., de Denus, S., & Lachaine, J. Systematic review of pharmacoeconomic studies of pharmacogenomic tests. *Pharmacogenomics* **11(11)**, 1573–1590 (2010)
4. Berm, E., de Looff, M., Wilffert, B., Boersma, C., Annemans ,L., Vegter, S., van Boven, J., Postma, M. Economic evaluations of pharmacogenetic and pharmacogenomic screening tests: A systematic review. Second update of the literature. *PLoS ONE* **11(1),** (2016)
5. Blank, P. R., Dedes, K. J., & Szucs, T. D. Cost effectiveness of cytotoxic and targeted therapy for metastatic breast cancer: A critical and systematic review. *PharmacoEconomics* **28(8),** 629-47 (2010)
6. Bongers, M. L., Coupe, V. M. H., Jansma, E. P., Smit, E. F., & Uyl-de Groot, C. A. Cost Effectiveness of Treatment With New Agents in Advanced Non-Small-Cell Lung Cancer A Systematic Review. *Pharmacoeconomics* **30(1),** 17–34 (2012)
7. Carlson, J. J., Henrikson, N. B., Veenstra, D. L., & Ramsey, S. D. Economic analyses of human genetics services: A systematic review. *Genetics in Medicine* ***7*(8),** 519–523 (2005)
8. Coate, L. E., & Leighl, N. B. How affordable are targeted therapies in non-small cell lung cancer? *Current Treatment Options in Oncology* **12(1),** 1–11 (2011)
9. D’Andrea, E., Marzuillo, C., De Vito, C., Di Marco, M., Pitini, E., Vacchio, M. R., & Villari, P. Which BRCA genetic testing programs are ready for implementation in health care? A systematic review of economic evaluations. *Genetics in Medicine* **18,** 1–10 (2016)
10. Degeling K. Koffijberg H. IJzerman M.J. A systematic review and checklist presenting the main challenges for health economic modeling in personalized medicine: towards implementing patient-level models. *Expert Review of Pharmacoeconomics and Outcomes Research* **17(1),** 17-25 (2017)
11. Djalalov, S., Musa, Z., Mendelson, M., Siminovitch, K., & Hoch, J. A review of economic evaluations of genetic testing services and interventions (2004–2009). *Genetics in Medicine* ***13*(2),** 89–94 (2011)
12. Doble, B., Tan, M., Harris, A., & Lorgelly, P. Modeling companion diagnostics in economic evaluations of targeted oncology therapies: systematic review and methodological checklist. *Expert Review of Molecular Diagnostics* **15(2),** 235–254 (2015)
13. Douglas, M. P., Ladabaum, U., Pletcher, M. J., Marshall, D. a., & Phillips, K. A. Economic evidence on identifying clinically actionable findings with whole-genome sequencing: a scoping review. *Genetics in Medicine* **18(2),** 1–6 (2016)
14. Ferrusi, I. L., Marshall, D. A., Kulin, N. A., Leighl, N. B., & Phillips, K. A. Looking back at 10 years of trastuzumab therapy: what is the role of HER2 testing? A systematic review of health economic analyses. *Personalized Medicine* **6(2),** 193–215 (2009)
15. Ferrusi, I. L., Leighl, N. B., Kulin, N. a, & Marshall, D. A. Do economic evaluations of targeted therapy provide support for decision makers? *Journal of Oncology Practice* **7(3 Suppl),** 36s–45s (2011)
16. Frank, M., Prenzler, A., Eils, R., Graf von der Schulenburg, J.-M., Lander, E., Linton, L., Ness, B. Van. Genome sequencing: a systematic review of health economic evidence. *Health Economics Review* **3(1),** 29 (2013a)
17. Frank, M., & Mittendorf, T. Influence of pharmacogenomic profiling prior to pharmaceutical treatment in metastatic colorectal cancer on cost effectiveness: A systematic review. *PharmacoEconomics* **31(3),** 215-28 (2013b)
18. Gavan, S., Harrison, M., Iglesias, C., Barton, A., Manca, A., & Payne, K. Economics of Stratified Medicine in Rheumatoid Arthritis. *Current Rheumatology Reports* **16(12),** 468 (2014)
19. Giacomini, M., Miller, F., & O’Brien, B. J. Economic considerations for health insurance coverage of emerging genetic tests. *Community Genetics* **6,** 61-73 (2003)
20. Gonzalez, F. M., Veneziano, M. A., Puggina, A., & Boccia, S. A Systematic Review on the Cost-Effectiveness of Genetic and Electrocardiogram Testing for Long QT Syndrome in Infants and Young Adults. *Value in Health* **18(5),** 700–708 (2015)
21. Griffith, G. L., Edwards, R. T., & Gray, J. Cancer genetics services: a systematic review of the economic evidence and issues. *British Journal of Cancer* **90(9),** 1697–703 (2004)
22. Hatz, M. H. M., Schremser, K., & Rogowski, W. H. Is individualized medicine more cost-effective? A systematic review. *PharmacoEconomics* **32(5),** 443-55 *(2014)*
23. Jarrett, J., & Mugford, M. (2006). Genetic health technology and economic evaluation: A critical review. *Applied Health Economics and Health Policy* **5(1),** 27-35 (2006)
24. Lange, A., Prenzler, A., Frank, M., Golpon, H., Welte, T., & von der Schulenburg, J.-M. A systematic review of the cost-effectiveness of targeted therapies for metastatic non-small cell lung cancer (NSCLC). *BMC Pulmonary Medicine* **14(1),** 192 (2014a)
25. Lange, A., Prenzler, A., Frank, M., Kirstein, M., Vogel, A., & Von Der Schulenburg, J. M. A systematic review of cost-effectiveness of monoclonal antibodies for metastatic colorectal cancer. *European Journal of Cancer* **50(1),** 40-49 (2014b)
26. Langer, A. A systematic review of PET and PET/CT in oncology: a way to personalize cancer treatment in a cost-effective manner? *BMC Health Services Research* **10,** 283 (2010)
27. Lansdorp-Vogelaar, I., Knudsen, A. B., & Brenner, H. Cost-effectiveness of colorectal cancer screening. *Epidemiologic Reviews* **33(1),** 88–100 (2011)
28. Lee, E., Illingworth, P., Wilton, L. & Chambers, G. M. The clinical effectiveness of preimplantation genetic diagnosis for aneuploidy in all 24 chromosomes (PGD-A): systematic review. *Human Reproduction*. 30, 473–48310 (2015).
29. Lieberthal, R. D. Economics of genomic testing for women with breast cancer. *The American Journal of Managed Care* **19(12),** 1024–31 (2013)
30. Meoni P. Economic evaluations of pharmacogenetic approaches in infectious diseases: A review of current approaches and evaluation of critical aspects affecting their quality. *Journal of Public Health in Africa* **4(2),** 78–83 (2013)
31. Nerich, V., Saing, S., Gamper, E. M., Kemmler, G., Daval, F., Pivot, X., & Holzner, B. Cost-utility analyses of drug therapies in breast cancer: a systematic review. *Breast Cancer Research and Treatment* **159(3),** 407-24 (2016)
32. Oosterhoff, M., van der Maas, M. E., & Steuten, L. M. G. A Systematic Review of Health Economic Evaluations of Diagnostic Biomarkers. *Applied Health Economics and Health Policy* **14(1),** 51-65 (2016)
33. Parkinson, B., Pearson, S.-A., & Viney, R. Economic evaluations of trastuzumab in HER2-positive metastatic breast cancer: a systematic review and critique. *The European Journal of Health Economics* **15(1),** 93-112 (2013)
34. Payne, K., Newman, W. G., Gurwitz, D., Ibarreta, D., & Phillips, K. A. TPMT testing in azathioprine: a “cost-effective use of healthcare resources”? *Personalized Medicine* **6(1),** 103–113 (2009)
35. Phillips, K. A, & Van Bebber, S. L. A systematic review of cost-effectiveness analyses of pharmacogenomic interventions. *Pharmacogenomics* **5(8),** 1139–1149 (2004)
36. Plöthner M, Ribbentrop D, Hartman J-P, Frank M. Cost-Effectiveness of Pharmacogenomic and Pharmacogenetic Test-Guided Personalized Therapies: A Systematic Review of the Approved Active Substances for Personalized Medicine in Germany. *Advances in Therapy* **33(9),** 1461-1480 (2016)
37. Plumpton, C. O., Roberts, D., Pirmohamed, M., & Hughes, D. A. A Systematic Review of Economic Evaluations of Pharmacogenetic Testing for Prevention of Adverse Drug Reactions. *PharmacoEconomics* **34(8),** 771-93 (2016)
38. Poonawalla, I. B., Parikh, R. C., Du, X. L., VonVille, H. M., & Lairson, D. R. Cost Effectiveness of Chemotherapeutic Agents and Targeted Biologics in Ovarian Cancer: A Systematic Review. *PharmacoEconomics* **33(11),** 1155-85 (2015)
39. Rogowski, W. Genetic screening by DNA technology: a systematic review of health economic evidence. *International Journal of Technology Assessment in Health Care* **22(3),** 327–337 (2006)
40. Smieliauskas, F., Chien, C. R., Shen, C., Geynisman, D. M., & Shih, Y. C. T. Cost-effectiveness analyses of targeted oral anti-cancer drugs: A systematic review. *PharmacoEconomics* **32(7),** 651-80 (2014)
41. [Stevanovic J](https://www.ncbi.nlm.nih.gov/pubmed/?term=Stevanovic%20J%5BAuthor%5D&cauthor=true&cauthor_uid=22988567)., [Postma MJ](https://www.ncbi.nlm.nih.gov/pubmed/?term=Postma%20MJ%5BAuthor%5D&cauthor=true&cauthor_uid=22988567), & [Pechlivanoglou P](https://www.ncbi.nlm.nih.gov/pubmed/?term=Pechlivanoglou%20P%5BAuthor%5D&cauthor=true&cauthor_uid=22988567). A systematic review on the application of cardiovascular risk prediction models in pharmacoeconomics, with a focus on primary prevention. [*European*](https://www.ncbi.nlm.nih.gov/pubmed/22988567) *Journal of Preventative Cardiology* **19(2),** 42-53 (2012)
42. Vegter, S., Boersma, C., Rozenbaum, M., Wilffert, B., Navis, G. J., & Postma, M. J. Pharmacoeconomic evaluations of pharmacogenetic and genomic screening programmes: A systematic review on content and adherence to guidelines. *PharmacoEconomics* **26(7),** 569-87 (2008)
43. Vegter, S., Jansen, E., Postma, M. J., & Boersma, C. Economic evaluations of pharmacogenetic and genomic screening programs: Update of the literature. *Drug Development Research* **71,** 492–501 (2010)
44. Verhoef, T. I., et al., A systematic review of cost-effectiveness analyses of pharmacogenetic-guided dosing in treatment with coumarin derivatives. *Pharmacogenomics* **11(7),** 989–1002 (2010)
45. Wong, W. B., Carlson, J. J., Thariani, R., & Veenstra, D. L. Cost effectiveness of pharmacogenomics: A critical and systematic review. *PharmacoEconomics* 28(11), 1001-13 (2010)
