# Supplementary material for: Accounting for Capacity Constraints in Economic Evaluations of Precision Medicine: A Systematic Review
Source: Pharmacoeconomics. 2019 May 13;37(8):1011–27. doi: 10.1007/s40273-019-00801-9 (PMC6597608; doi:10.1007/s40273-019-00801-9)
Supplement: Supplementary file 3 — Supplementary material 3 (DOCX 37 kb) [file 40273_2019_801_MOESM3_ESM.docx]

PharmacoEconomics. Accounting for Capacity Constraints in Economic Evaluations of Precision Medicine: a Systematic Review. Stuart J Wright, William Newman, Katherine Payne

Correspondence to Stuart J Wright, Manchester Centre for Health Economics, Division of Population Health, Health Services Research & Primary Care, The University of Manchester, Oxford Road, Manchester, M13 9PL, stuart.wright-2@manchester.ac.uk, 01613067970

**Supplementary Appendix 3 – References of 222 Included Studies**

Aboutorabi A, Hadian M, Ghaderi H, Salehi M, Ghiasipour M. Cost-effectiveness analysis of trastuzumab in the adjuvant treatment for early breast cancer. Glob. J. Health Sci. [Internet]. 2015;7:98–106.

Asseburg C, Frank M, Kohne C-H, Hartmann JT, Griebsch I, Mohr A, et al. Cost-effectiveness of targeted therapy with cetuximab in patients with K-ras wild-type colorectal cancer presenting with initially unresectable metastases limited to the liver in a German setting. Clin. Ther. [Internet]. United States; 2011;33:482–97.

Athanasakis K, Kyriopoulos J. A cost-effectiveness analysis of trastuzumab plus docetaxal vs. docetaxal alone for the treatment of HER2-positive metastatic breast cancer in the Greek healthcare setting. Forum Clin. Oncol. 2012;3. 4.

Atherly AJ, Camidge DR. The cost-effectiveness of screening lung cancer patients for targeted drug sensitivity markers. Br. J. Cancer [Internet]. England; 2012;106:1100–6.

Attard CL, Pepper a N, Brown ST, Thompson MF, Thuresson P-O, Yunger S, et al. Cost-effectiveness analysis of neoadjuvant pertuzumab and trastuzumab therapy for locally advanced, inflammatory, or early HER2-positive breast cancer in Canada. J. Med. Econ. [Internet]. 2014;1–16.

Au HJ, Golmohammadi K, Younis T, Verma S, Chia S, Fassbender K, et al. Cost-effectiveness analysis of adjuvant docetaxel, doxorubicin, and cyclophosphamide (TAC) for node-positive breast cancer: Modeling the downstream effects. Breast Cancer Res. Treat. 2009;114:579–87. 7.

Bacchi CE, Prisco F, Carvalho FM, Ojopi EB, Saad ED. Potential economic impact of the 21-gene expression assay on the treatment of breast cancer in Brazil. Rev. Assoc. Med. Bras. [Internet]. Brazil; 2010;56:186–91.

Barone C, Pinto C, Normanno N, Capussotti L, Cognetti F, Falcone A, et al. KRAS early testing: Consensus initiative and cost-effectiveness evaluation for metastatic colorectal patients in an italian setting. PLoS One. 2014;9. 9.

Behl AS, Goddard KAB, Flottemesch TJ, Veenstra D, Meenan RT, Lin JS, et al. Cost-effectiveness analysis of screening for KRAS and BRAF mutations in metastatic colorectal cancer. J. Natl. Cancer Inst. 2012;104:1785–95. 10.

Bird A, Norman R, Goodall S. Economic Evaluation of Positron Emission Tomography (PET) in Non Small Cell Lung Cancer (NSCLC). Sydney; 2007. Report No.: 2007/6. 11.

Blank PR, Moch H, Szucs TD, Schwenkglenks M. KRAS and BRAF mutation analysis in metastatic colorectal cancer: A cost-effectiveness analysis from a Swiss perspective. Clin. Cancer Res. 2011;17:6338–46. 12.

Blank PR, Schwenkglenks M, Moch H, Szucs TD. Human epidermal growth factor receptor 2 expression in early breast cancer patients: A Swiss cost-effectiveness analysis of different predictive assay strategies. Breast Cancer Res. Treat. 2010;124:497–507. 13.

Blohmer JU, Rezai M, Kümmel S, Kühn T, Warm M, Friedrichs K, et al. Using the 21-gene assay to guide adjuvant chemotherapy decision-making in early-stage breast cancer: a cost-effectiveness evaluation in the German setting. J. Med. Econ. [Internet]. 2013;16:30–40.

Borget I, Cadranel J, Pignon J-P, Quoix E, Coudert B, Westeel V, et al. Cost-effectiveness of three strategies for second-line erlotinib initiation in nonsmall-cell lung cancer: the ERMETIC study part 3. Eur. Respir. J. 2012;39:172–9. 15.

Bradbury PA, Tu D, Seymour L, Isogai PK, Zhu L, Ng R, et al. Economic Analysis: Randomized Placebo-Controlled Clinical Trial of Erlotinib in Advanced Non-Small Cell Lung Cancer. JNCI J. Natl. Cancer Inst. [Internet]. Oxford University Press; 2010 [cited 2017 Jan 31];102:298–306.

Braun S, Mittendorf T, Menschik T, Greiner W, Von Der Schulenburg JM. Cost effectiveness of exemestane versus tamoxifen in post-menopausal women with early breast cancer in Germany. Breast Care. 2009;4:389–96. 17.

Breijer MC, Van Doorn HC, Clark TJ, Khan KS, Timmermans A, Mol BWJ, et al. Diagnostic strategies for endometrial cancer in women with postmenopausal bleeding: Cost-effectiveness of individualized strategies. Eur. J. Obstet. Gynecol. Reprod. Biol. [Internet]. Elsevier Ireland Ltd; 2012;163:91–6.

Buendia JA, Vallejos C, Pichon-Riviere A. An economic evaluation of trastuzumab as adjuvant treatment of early HER2-positive breast cancer patients in Colombia. Biomedica. 2013;33:411–7. 19.

Cameron DA, Camidge DR, Oyee J, Hirsch M. Economic evaluation of fulvestrant as an extra step in the treatment sequence for ER-positive advanced breast cancer. Br. J. Cancer [Internet]. 2008;99:1984–90.

Camma C, Petta S, Enea M, Bruno R, Bronte F, Capursi V, et al. Cost-effectiveness of sofosbuvir-based triple therapy for untreated patients with genotype 1 chronic hepatitis C. Hepatology. 2014;59:1692–705. 21.

Candon D, Healy J, Crown J. Modelling the cost-effectiveness of adjuvant lapatinib for early-stage breast cancer. Acta Oncol. [Internet]. 2014;53:201–8.

Carlson JJ, Garrison LP, Ramsey SD, Veenstra DL. The Potential Clinical and Economic Outcomes of Pharmacogenomic Approaches to EGFR-Tyrosine Kinase Inhibitor Therapy in Non–Small-Cell Lung Cancer. Value Heal. [Internet]. 2009 [cited 2017 Jan 31];12:20–7.

Caro JJ, Stillman IO, Danel A, Getsios D, McEwan P. Cost effectiveness of rimonabant use in patients at increased cardiometabolic risk: Estimates from a Markov model. J. Med. Econ. [Internet]. 2007;10:239–54.

Chen A, Dowdy DW. Clinical effectiveness and cost-effectiveness of HIV pre-exposure prophylaxis in men who have sex with men: Risk calculators for real-world decision-making. PLoS One. 2014;9. 25.

Chen E, Tong KB, Malin JL. Cost-Effectiveness of 70-Gene MammaPrint Signature in Node-Negative Breast Cancer. Am. J. Manag. Care. 2010;16:333–42. 26.

Chen W, Jiang Z, Shao Z, Sun Q, Shen K. An economic evaluation of adjuvant trastuzumab therapy in HER2-positive early breast cancer. Value Heal. [Internet]. International Society for Pharmacoeconomics and Outcomes Research (ISPOR); 2009;12:S82–4.

Cheng TF, Wang J Der, Uen WC. Cost-utility analysis of adjuvant goserelin (Zoladex) and adjuvant chemotherapy in premenopausal women with breast cancer. BMC Cancer [Internet]. BioMed Central Ltd; 2012;12:33.

Chouaid C, Monnet I, Robinet G, Perol M, Fournel P, Vergnenegre A. Economic impact of gefitinib for refractory non-small-cell lung cancer: a Markov model-based analysis. Curr. Med. Res. Opin. [Internet]. England; 2007;23:1509–15.

Collinson P, Gaze D, Goodacre S, Bradburn M. RATPAC CBE (Randomised Assessment of Treatment using Panel Assay of Cardiac markers - Contemporary Biomarker Evaluation). Health Technol. Assess. (Rockv). 2013;17:1–17. 30.

Contreras-Hernández I, Mould-Quevedo JF, Silva A, Salinas-Escudero G, Villasís-Keever MA, Granados-García V, et al. A pharmaco-economic analysis of second-line treatment with imatinib or sunitinib in patients with advanced gastrointestinal stromal tumours. Br. J. Cancer [Internet]. 2008;98:1762–8.

Contreras-Hernandez I, Becker D, Chancellor J, Kühne F, Mould-Quevedo J, Vega G, et al. Cost-effectiveness of maraviroc for antiretroviral treatment-experienced HIV-infected individuals in Mexico. Value Heal. [Internet]. International Society for Pharmacoeconomics and Outcomes Research (ISPOR); 2010;13:903–14.

Cosler LE, Lyman GH. Economic analysis of gene expression profile data to guide adjuvant treatment in women with early-stage breast cancer. Cancer Invest. [Internet]. 2009;27:953–9.

Costa-Scharplatz M, van Asselt ADI, Bachmann LM, Kessels AGH, Severens JL. Cost-effectiveness of pharmacogenetic testing to predict treatment response to angiotensin-converting enzyme inhibitor. Pharmacogenet. Genomics [Internet]. United States; 2007;17:359–68.

Crespin DJ, Federspiel JJ, Biddle AK, Jonas DE, Rossi JS. Ticagrelor versus genotype-driven antiplatelet therapy for a secondary prevention after acute coronary syndrome: a cost-effectiveness analysis. Value Heal. 2011;14:483–91. 35.

Das R, Cope S, Ouwens M, Turner P, Howlett M. Economic Evaluation of Fulvestrant 500 mg Versus Generic Nonsteroidal Aromatase Inhibitors in Patients With Advanced Breast Cancer in the United Kingdom. Clin. Ther. [Internet]. Elsevier Inc.; 2013;35:246–260.e5.

Davidson JA, Cromwell I, Ellard SL, Lohrisch C, Gelmon KA, Shenkier T, et al. A prospective clinical utility and pharmacoeconomic study of the impact of the 21-gene Recurrence Score?? assay in oestrogen receptor positive node negative breast cancer. Eur. J. Cancer [Internet]. Elsevier Ltd; 2013;49:2469–75.

de Lima Lopes G, Segel JE, Tan DSW, Do YK, Mok T, Finkelstein EA. Cost-effectiveness of epidermal growth factor receptor mutation testing and first-line treatment with gefitinib for patients with advanced adenocarcinoma of the lung. Cancer. 2012;118:1032–9. 38.

Dedes KJ, Matter-Walstra K, Schwenkglenks M, Pestalozzi BC, Fink D, Brauchli P, et al. Bevacizumab in combination with paclitaxel for HER-2 negative metastatic breast cancer: An economic evaluation. Eur. J. Cancer [Internet]. Elsevier Ltd; 2009;45:1397–406.

Dedes KJ, Szucs TD, Imesch P, Fedier A, Fehr MK, Fink D. Cost-effectiveness of trastuzumab in the adjuvant treatment of early breast cancer: A model-based analysis of the HERA and FinHer trial. Ann. Oncol. 2007;18:1493–9. 40.

Delea TE, Amdahl J, Chit A, Amonkar MM. Cost-effectiveness of lapatinib plus letrozole in HER2-positive, hormone receptor-positive metastatic breast cancer in Canada. Curr. Oncol. 2013;20:371–87. 41.

Delea TE, El-Ouagari K, Karnon J, Sofrygin O. Cost-effectiveness of letrozole versus tamoxifen as initial adjuvant therapy in postmenopausal women with hormone-receptor positive early breast cancer from a Canadian perspective. Breast Cancer Res. Treat. 2008;108:375–87. 42.

Delea TE, Hawkes C, Amonkar MM, Lykopoulos K, Johnston SRD. Cost-effectiveness of lapatinib plus letrozole in post-menopausal women with hormone receptor- and HER2-positive metastatic breast cancer. Breast Care. 2013;8:429–37. 43.

Delea TE, Karnon J, Sofrygin O, Thomas SK, Papo NL, Barghout V. Cost-Effectiveness of Letrozole Versus Tamoxifen as Initial Adjuvant Therapy in Hormone Receptor–Positive Postmenopausal Women with Early-Stage Breast Cancer. Clin. Breast Cancer [Internet]. Elsevier Inc.; 2007;7:608–18.

Delea TE, Taneja C, Sofrygin O, Kaura S, Gnant M. Cost-Effectiveness of Zoledronic Acid Plus Endocrine Therapy in Premenopausal Women With Hormone-Responsive Early Breast Cancer. Clin. Breast Cancer [Internet]. Elsevier Inc.; 2010;10:267–74.

Delea TE, Tappenden P, Sofrygin O, Browning D, Amonkar MM, Karnon J, et al. Cost-effectiveness of lapatinib plus capecitabine in women with HER2+ metastatic breast cancer who have received prior therapy with trastuzumab. Eur. J. Heal. Econ. 2012;13:589–603. 46.

Dendukuri N, Khetani K, McIsaac M, Brophy J. Testing for HER2-positive breast cancer: a systematic review and cost-effectiveness analysis. CMAJ [Internet]. Canada; 2007;176:1429–34.

Diaby V, Adunlin G, Zeichner SB, Avancha K, Lopes G. Cost-effectiveness analysis of everolimus plus exemestane versus exemestane alone for treatment of hormone receptor positive breast cancer. Breast Cancer Res. Treat. 2014;147:433–41. 48.

Dionne F, Mitton C, Rassekh R, Brooks B, Ross C, Hayden M, et al. Economic impact of a genetic test for cisplatin-induced ototoxicity. Pharmacogenomics J. [Internet]. United States; 2012;12:205–13.

Djalalov S, Beca J, Hoch JS, Krahn M, Tsao M-S, Cutz J-C, et al. Cost effectiveness of EML4-ALK fusion testing and first-line crizotinib treatment for patients with advanced ALK-positive non-small-cell lung cancer. J. Clin. Oncol. [Internet]. United States; 2014;32:1012–9..

Djalalov S, Yong J, Beca J, Black S, Saposnik G, Musa Z, et al. Genetic Testing in Combination with Preventive Donepezil Treatment for Patients with Amnestic Mild Cognitive Impairment. Mol. Diagn. Ther. [Internet]. Springer International Publishing AG; 2012 [cited 2017 Sep 19];16:389–99.

Dong D, Sung C, Finkelstein A. Cost-effectiveness of HLA-B*1502 genotyping in adult patients with newly diagnosed epilepsy in Singapore. Neurology. 2013;80:1621–2. 52.

Donnan JR, Ungar WJ, Matthews M, Hancock-Howard RL, Rahman P. A Cost Effectiveness Analysis of Thiopurine Methyltransferase Testing for Guiding 6-Mercaptopurine Dosing in Children With Acute Lymphoblastic Leukemia. Pediatr. Blood Cancer. 2011;57:231–9. 53.

Duarte A, Burch J, Smith A, Walker S, Fox D, Rodriguez-Lopez, et al. Crizotinib for ALK fusion positive NSCLC : ERG report Crizotinib for ALK fusion positive NSCLC : ERG report. York; 2013. 54.

Ebara T, Ohno T, Nakano T. Quantitative medical cost-effectiveness analysis of molecular-targeting cancer drugs in Japan. DARU J. Fac. Pharm. Sci. [Internet]. 2013;21:40.

Eckman MH, Rosand J, Greenberg SM, Gage BF. Cost-effectiveness of using pharmacogenetic information in warfarin dosing for patients with nonvalvular atrial fibrillation. Ann. Intern. Med. 2009;150. 56.

El Ouagari K, Karnon J, Delea T, Talbot W, Brandman J. Cost-effectiveness of letrozole in the extended adjuvant treatment of women with early breast cancer. Breast Cancer Res. Treat. 2007;101:37–49. 57.

Erman A, Nugent A, Amir E, Coyte PC. Cost-effectiveness analysis of extended adjuvant endocrine therapy in the treatment of post-menopausal women with hormone receptor positive breast cancer. Breast Cancer Res. Treat. 2014;145:267–79. 58.

Essers BAB, Seferina SC, Tjan-Heijnen VCG, Severens JL, Novák A, Pompen M, et al. Transferability of model-based economic evaluations: The case of trastuzumab for the adjuvant treatment of her2-positive early breast cancer in the netherlands. Value Heal. [Internet]. International Society for Pharmacoeconomics and Outcomes Research (ISPOR); 2010;13:375–80.

Fitzgerald P, Goodacre SW, Cross E, Dixon S. Cost-effectiveness of point-of-care biomarker assessment for suspected myocardial infarction: the randomized assessment of treatment using panel Assay of cardiac markers (RATPAC) trial. Acad. Emerg. Med. [Internet]. United States; 2011;18:488–95.

Fleeman N, NIHR Health Technology Assessment Programme (Great Britain), NIHR Evaluation Trials and Studies Coordinating Centre (Great Britain). Lapatinib and trastuzumab in combination with an aromatase inhibitor for the first-line treatment of metastatic hormone receptor-positive breast cancer which over-expresses human epidermal growth factor 2 (HER2) : a systematic review and economic analysis. Heal. Technol. assessment,. 2011;15:xvi, 100 p. 61.

Furuta T, Shirai N, Kodaira M, Sugimoto M, Nogaki A, Kuriyama S, et al. Pharmacogenomics-based tailored versus standard therapeutic regimen for eradication of H. pylori. Clin. Pharmacol. Ther. [Internet]. United States; 2007;81:521–8.

Gani Ms, Shafee A, Soliman I. Ultrasound guided percutaneous fine needle aspiration biopsy / automated needle core biopsy of abdominal lesions: Effect on management and cost effectiveness. Ann. Afr. Med. [Internet]. 2011;10:133.

Garrison LP, Lalla D, Brammer M, Babigumira JB, Wang B, Perez EA. Assessing the potential cost-effectiveness of retesting IHC0, IHC1+, or FISH-negative early stage breast cancer patients for HER2 status. Cancer. 2013;119:3113–22. 64.

Garrison LP, Lubeck D, Lalla D, Paton V, Dueck A, Perez EA. Cost-effectiveness analysis of trastuzumab in the adjuvant setting for treatment of HER2-positive breast cancer. Cancer. 2007;110:489–98. 65.

Garrison LP, Veenstra DL. The economic value of innovative treatments over the product life cycle: The case of targeted trastuzumab therapy for breast cancer. Value Heal. [Internet]. International Society for Pharmacoeconomics and Outcomes Research (ISPOR); 2009;12:1118–23.

Gausachs M, Mur P, Corral J, Pineda M, González S, Benito L, et al. MLH1 promoter hypermethylation in the analytical algorithm of Lynch syndrome: a cost-effectiveness study. Eur. J. Hum. Genet. 2012;20:762–8. 67.

Gold HT, Hall MJ, Blinder V, Schackman BR. Cost effectiveness of pharmacogenetic testing for UGT1A1 before irinotecan administration for metastatic colorectal cancer. 2009;115:3858–67. 68.

Gordon LG, Hirst NG, Young RP, Brown PM. Within a smoking-cessation program, what impact does genetic information on lung cancer need to have to demonstrate cost-effectiveness? Cost Eff. Resour. Alloc. [Internet]. 2010;8:18.

Greeley SAW, John PM, Winn AN, Ornelas J, Lipton RB, Philipson LH, et al. The cost-effectiveness of personalized genetic medicine: the case of genetic testing in neonatal diabetes. Diabetes Care [Internet]. United States; 2011;34:622–7.

Hagaman JT, Kinder BW, Eckman MH. Thiopurine S-methyltranferase testing in idiopathic pulmonary fibrosis: A pharmacogenetic cost-effectiveness analysis. Lung. 2010;188. 71.

Hall PS, McCabe C, Stein RC, Cameron D. Economic evaluation of genomic test-directed chemotherapy for early-stage lymph node-positive breast cancer. J. Natl. Cancer Inst. 2012;104:56–66. 72.

Hall PS, Hulme C, Mccabe C, Oluboyede Y, Round J, Cameron D a. Updated Cost-Effectiveness Analysis of Trastuzumab for Early Breast Cancer Toxicity and Pattern of Recurrence. 2011;29:415–32. 73.

Handorf EA, Mcelligott S, Vachani A, Langer CJ, Demeter B, Armstrong K, et al. Cost Effectiveness of Personalized Therapy for First-Line Treatment of Stage IV and Recurrent Incurable Adenocarcinoma of the Lung. 74.

Hannouf MB, Xie B, Brackstone M, Zaric GS. Cost effectiveness of a 21-gene recurrence score assay versus canadian clinical practice in post-menopausal women with early-stage estrogen or progesterone-receptor-positive, axillary lymph-node positive breast cancer. Pharmacoeconomics. 2014;32:135–47. 75.

Hannouf MB, Xie B, Brackstone M, Zaric GS. Cost-effectiveness of a 21-gene recurrence score assay versus Canadian clinical practice in women with early-stage estrogen- or progesterone- receptor-positive , axillary lymph-node negative breast cancer. BMC Cancer [Internet]. 2012;12:1. Available from: BMC Cancer76.

Hedden L, O’Reilly S, Lohrisch C, Chia S, Speers C, Kovacic L, et al. Assessing the real-world cost-effectiveness of adjuvant trastuzumab in HER-2/neu positive breast cancer. Oncologist [Internet]. 2012;17:164–71.

Heilbrun ME, Yu J, Smith KJ, Dechet CB, Zagoria RJ, Roberts MS. The Cost-Effectiveness of Immediate Treatment, Percutaneous Biopsy and Active Surveillance for the Diagnosis of the Small Solid Renal Mass: Evidence From a Markov Model. J. Urol. [Internet]. Elsevier; 2012 [cited 2018 Aug 23];187:39–43.

Heitjan DF, Asch D a, Ray R, Rukstalis M, Patterson F, Lerman C. Cost-effectiveness of pharmacogenetic testing to tailor smoking-cessation treatment. Pharmacogenomics J. [Internet]. 2008;8:391–9.

Heller M, Zanocco K, Zydowicz S, Elaraj D, Nayar R, Sturgeon C. Cost-effectiveness analysis of repeat fine-needle aspiration for thyroid biopsies read as atypia of undetermined significance. Surg. (United States) [Internet]. Mosby, Inc.; 2012;152:423–30.

Holt S, Bertelli G, Humphreys I, Valentine W, Durrani S, Pudney D, et al. A decision impact, decision conflict and economic assessment of routine Oncotype DX testing of 146 women with node-negative or pNImi, ER-positive breast cancer in the UK. Br. J. Cancer [Internet]. Nature Publishing Group; 2013;108:2250–8.

Hornberger J, Chien R, Krebs K, Hochheiser L. US insurance program’s experience with a multigene assay for early-stage breast cancer. Am. J. Manag. Care. 2011;17. 82.

Hoyle M, Peters J, Crathorne L, Jones-Hughes T, Cooper C, Napier M, et al. Cost-effectiveness of cetuximab, cetuximab plus irinotecan, and panitumumab for third and further lines of treatment for KRAS wild-type patients with metastatic colorectal cancer. Value Heal. [Internet]. Elsevier; 2013;16:288–96.

Ito K, Elkin E, Blinder V, Keating N, Choudhry N. Cost-effectiveness of full coverage of aromatase inhibitors for Medicare beneficiaries with early breast cancer. Cancer. 2013;119:2494–502. 84.

Kapoor R, Martinez-Vega R, Dong D, Tan SY, Leo Y-S, Lee C-C, et al. Reducing hypersensitivity reactions with HLA-B*5701 genotyping before abacavir prescription: clinically useful but is it cost-effective in Singapore? Pharmacogenet. Genomics [Internet]. 2015;25:60–72.

Karnon J, Delea T, Barghout V. Cost utility analysis of early adjuvant letrozole or anastrozole versus tamoxifen in postmenopausal women with early invasive breast cancer: the UK perspective. Eur. J. Health Econ. [Internet]. 2008;9:171–83.

Kauf TL, Farkouh RA, Earnshaw SR, Watson ME, Maroudas P, Chambers MG. Economic efficiency of genetic screening to inform the use of abacavir sulfate in the treatment of HIV. Pharmacoeconomics. 2010;28:1025–39. 87.

Kazi DS, Garber AM, Shah RU, Dudley RA, Mell MW. Original Research Cost-Effectiveness of Genotype-Guided and Dual Antiplatelet Therapies. 2014; 88.

Kee F, Erridge S, Bradbury I, Cairns K. The value of positron emission tomography in patients with non-small cell lung cancer. Eur. J. Radiol. 2010;73:50–8. 89.

Klang SH, Hammerman A, Liebermann N, Efrat N, Doberne J, Hornberger J. Economic implications of 21-gene breast cancer risk assay from the perspective of an Israeli-managed health-care organization. Value Heal. [Internet]. International Society for Pharmacoeconomics and Outcomes Research (ISPOR); 2010;13:381–7.

Kondo M, Hoshi SL, Ishiguro H, Yoshibayashi H, Toi M. Economic evaluation of 21-gene reverse transcriptase-polymerase chain reaction assay in lymph-node-negative, estrogen-receptor-positive, early-stage breast cancer in Japan. Breast Cancer Res. Treat. 2008;112:175–87. 91.

Kondo M, Hoshi SL, Yamanaka T, Ishiguro H, Toi M. Economic evaluation of the 21-gene signature (Oncotype DX) in lymph node-negative/positive, hormone receptor-positive early-stage breast cancer based on Japanese validation study (JBCRG-TR03). Breast Cancer Res. Treat. 2011;127:739–49. 92.

Kondo M, Hoshi S-L, Ishiguro H, Toi M. Economic evaluation of the 70-gene prognosis-signature (MammaPrint®) in hormone receptor-positive, lymph node-negative, human epidermal growth factor receptor type 2-negative early stage breast cancer in Japan. Breast Cancer Res. Treat. [Internet]. 2012;133:759–68.

Konnopka A, Conrad K, Baerwald C, Konig HH. Cost effectiveness of the determination of autoantibodies against cyclic citrullinated peptide in the early diagnosis of rheumatoid arthritis. Ann Rheum Dis [Internet]. 2008;67:1399–405.

Kowada A. Cost effectiveness of interferon-gamma release assay for tuberculosis screening of rheumatoid arthritis patients prior to initiation of tumor necrosis factor-alpha antagonist therapy. Mol. Diagn. Ther. 2010;14:367–73. 95.

Krieckaert CLM, Nair SC, Nurmohamed MT, van Dongen CJJ, Lems WF, Lafeber FPJG, et al. Personalised treatment using serum drug levels of adalimumab in patients with rheumatoid arthritis: an evaluation of costs and effects. Ann. Rheum. Dis. [Internet]. 2013;361–8.

Krug B, Crott R, Roch I, Lonneux M, Beguin C, Baurain JF, et al. Cost-effectiveness analysis of FDG PET-CT in the management of pulmonary metastases from malignant melanoma. Acta Oncol. (Madr). 2010;49:192–200. 97.

Kühne F, Chancellor J, Mollon P, Myers DE, Louise M, Powderly WG. A Microsimulation of the Cost-effectiveness of Maraviroc for Antiretroviral Treatment- Experienced HIV-Infected Individuals. HIV Clin. Trials. 2015;11:80–99. 98.

Kurian AW, Thompson RN, Gaw AF, Arai S, Ortiz R, Garber AM. A cost-effectiveness analysis of adjuvant trastuzumab regimens in early HER2/neu-positive breast cancer. J. Clin. Oncol. 2007;25:634–41. 99.

Kwon JS, Daniels MS, Sun CC, Lu KH. Preventing future cancers by testing women with ovarian cancer for BRCA mutations. J. Clin. Oncol. 2010;28:675–82. 100.

Lala A, Berger JS, Sharma G, Hochman JS, Scott Braithwaite R, Ladapo JA. Genetic testing in patients with acute coronary syndrome undergoing percutaneous coronary intervention: a cost-effectiveness analysis. J. Thromb. Haemost. [Internet]. England; 2013;11:81–91.

Lamond NWD, Skedgel C, Rayson D, Lethbridge L, Younis T. Cost-utility of the 21-gene recurrence score assay in node-negative and node-positive breast cancer. Breast Cancer Res. Treat. 2012;133:1115–23. 102.

Le QA, Hay JW. Cost-effectiveness analysis of lapatinib in HER-2-positive advanced breast cancer. Cancer. 2009;115:489–98. 103.

Lee H-J, Lee T-J, Yang B-M, Min J. Cost-Effectiveness Analysis of Adjuvant Hormonal Treatments for Women with Postmenopausal Hormone-Receptor Positive Early Breast Cancer in the Korean Context. J. Breast Cancer [Internet]. 2010 [cited 2018 Aug 23];13:286.

Lee SG, Jee YG, Chung HC, Kim SB, Ro J, Im YH, et al. Cost-effectiveness analysis of adjuvant therapy for node positive breast cancer in Korea: Docetaxel, doxorubicin and cyclophosphamide (TAC) versus fluorouracil, doxorubicin and cyclophosphamide (FAC). Breast Cancer Res. Treat. 2009;114:589–95. 105.

Leey JA, McCabe S, Koch JA, Miles TP. Cost-effectiveness of genotype-guided warfarin therapy for anticoagulation in elderly patients with atrial fibrillation. Am. J. Geriatr. Pharmacother. [Internet]. Elsevier; 2009 [cited 2018 Aug 23];7:197–203.

Li H, Robinson KA, Anton B, Saldanha IJ, Ladenson PW. Cost-effectiveness of a novel molecular test for cytologically indeterminate thyroid nodules. J. Clin. Endocrinol. Metab. 2011;96:1719–26. 107.

Liberato NL, Marchetti M, Barosi G. Cost effectiveness of adjuvant trastuzumab in human epidermal growth factor receptor 2-positive breast cancer. J. Clin. Oncol. 2007;25:625–33. 108.

Lidgren M, Jönsson B, Rehnberg C, Willking N, Bergh J. Cost-effectiveness of HER2 testing and 1-year adjuvant trastuzumab therapy for early breast cancer. Ann. Oncol. 2008;19:487–95. 109.

Lidgren M, Wilking N, Jonsson B, Rehnberg C. Cost-effectiveness of HER2 testing and trastuzumab therapy for metastatic breast cancer. Acta Oncol. (Madr). 2008;47:1018–28. 110.

Lieberthal RD, Dudash K, Axelrod R, Goldfarb N. An economic model to value companion diagnostics in non-small-cell lung cancer. Per. Med. 2013;10:139–47. 111.

Lipsitz M, Delea TE, Guo A. Cost effectiveness of letrozole versus anastrozole in postmenopausal women with HR+ early-stage breast cancer. Curr. Med. Res. Opin. [Internet]. 2010;26:2315–28.

Liu S, Cipriano L, Holodniy M, Owens D, Goldhaber-fiebert J. New Protease Inhibitors for the Treatment of Chronic Hepatitis C: A Cost-Effectiveness Analysis. Ann. Intern. Med. 2012;156:279–90. 113.

Liu S, Schwarzinger M, Carrat F, Goldhaber-Fiebert JD. Cost effectiveness of fibrosis assessment prior to treatment for chronic hepatitis C patients. PLoS One [Internet]. United States; 2011;6:e26783.

Locker GY, Mansel R, Cella D, Dobrez D, Sorensen S, Gandhi SK. Cost-effectiveness analysis of anastrozole versus tamoxifen as primary adjuvant therapy for postmenopausal women with early breast cancer: A US healthcare system perspective. The 5-year completed treatment analysis of the ATAC (“Arimidex”, Tamoxifen Alone. Breast Cancer Res. Treat. 2007;106:229–38. 115.

Lorenzana SB, Hughes MD, Grinsztejn B, Collier AC, Luz PM, Freedberg KA, et al. Genotype assays and third-line ART in resource-limited settings: A simulation and cost-effectiveness analysis of a planned clinical trial. Aids. 2012;26:1083–93. 116.

Lundkvist J, Wilking N, Holmberg S, Jönsson L. Cost-effectiveness of exemestane versus tamoxifen as adjuvant therapy for early-stage breast cancer after 2-3 years treatment with tamoxifen in Sweden. Breast Cancer Res. Treat. 2007;102:289–99. 117.

Lux MP, Reichelt C, Karnon J, Tänzer TD, Radosavac D, Fasching PA, et al. Cost-benefit analysis of endocrine therapy in the adjuvant setting for postmenopausal patients with hormone receptor-positive breast cancer, based on survival data and future prices for generic drugs in the context of the German health care system. Breast Care. 2011;6:381–9. 118.

Lux MP, Wöckel A, Benedict A, Buchholz S, Kreif N, Harbeck N, et al. Cost-effectiveness analysis of anastrozole versus tamoxifen in adjuvant therapy for early-stage breast cancer - A health-economic analysis based on the 100-month analysis of the atac trial and the German health system. Onkologie. 2010;33:155–66. 119.

Lux MP, Hartmann M, Jackisch C, Raab G, Schneeweiß A, Possinger K, et al. Cost-utility analysis for advanced breast cancer therapy in Germany: Results of the fulvestrant sequencing model. Breast Cancer Res. Treat. 2009;117:305–17. 120.

Lyman GH, Cosler LE, Kuderer NM, Hornberger J. Impact of a 21-gene RT-PCR assay on treatment decisions in early-stage breast cancer: An economic analysis based on prognostic and predictive validation studies. Cancer. 2007;109:1011–8. 121.

Mabasa VH, Taylor SC, Chu CC, Moravan V, Johnston K, Peacock S, et al. Verification of imatinib cost-effectiveness in advanced gastrointestinal stromal tumor in British Columbia (VINCE-BC study). J Oncol Pharm Pr. [Internet]. 2008;14:105–12.

Machado M, Einarson TR. Lapatinib in patients with metastatic breast cancer following initial treatment with trastuzumab: an economic analysis from the Brazilian public health care perspective. Breast cancer (Dove Med. Press. [Internet]. 2012;4:173–82.

Majer IM, Gelderblom H, van den Hout WB, Gray E, Verheggen BG. Cost-effectiveness of 3-year vs 1-year adjuvant therapy with imatinib in patients with high risk of gastrointestinal stromal tumour recurrence in the Netherlands; a modelling study alongside the SSGXVIII/AIO trial. J. Med. Econ. [Internet]. 2013;16:1106–19.

Manchanda R, Patel S, Antoniou AC, Levy-Lahad E, Turnbull C, Evans DG, et al. Cost-effectiveness of population based BRCA testing with varying Ashkenazi Jewish ancestry. Am. J. Obstet. Gynecol. [Internet]. United States; 2017;217:578.e1-578.e12.

Mansel R, Locker G, Fallowfield L, Benedict Á, Jones D. Cost-effectiveness analysis of anastrozole vs tamoxifen in adjuvant therapy for early stage breast cancer in the United Kingdom: The 5-year completed treatment analysis of the ATAC (“Arimidex”, Tamoxifen alone or in combination) trial. Br. J. Cancer [Internet]. 2007;97:152–61.

Mansueto M, Grimaldi A, Mangili G, Picchio M, Giovacchini G, Vigan?? R, et al. Positron emission tomography/computed tomography introduction in the clinical management of patients with suspected recurrence of ovarian cancer: A cost-effectiveness analysis. Eur. J. Cancer Care (Engl). 2009;18:612–9. 127.

Mansueto M, Grimaldi A, Torbica A, Pepe G, Giovacchini G, Messa C, et al. Cost-effectiveness analysis in the clinical management of patients with known or suspected lung cancer: [18F]fluorodeoxyglucose PET and CT comparison. Q. J. Nucl. Med. Mol. Imaging. 2007;51:224–34. 128.

Marino P, Siani C, Roch?? H, Proti??re C, Fumoleau P, Spielmann M, et al. Cost-effectiveness of adjuvant docetaxel for node-positive breast cancer patients: Results of the PACS 01 economic study. Ann. Oncol. 2010;21:1448–54. 129.

Marino P, Siani C, Bertucci F, Roche H, Martin AL, Viens P, et al. Economic issues involved in integrating genomic testing into clinical care: The case of genomic testing to guide decision-making about chemotherapy for breast cancer patients. Breast Cancer Res. Treat. 2011;129:401–9. 130.

Matter-Walstra KW, Dedes KJ, Schwenkglenks M, Brauchli P, Szucs TD, Pestalozzi BC. Trastuzumab beyond progression: A cost-utility analysis. Ann. Oncol. 2010;21:2161–8. 131.

McCowan C, Wang S, Thompson AM, Makubate B, Petrie DJ. The value of high adherence to tamoxifen in women with breast cancer: a community-based cohort study. Br J Cancer [Internet]. Nature Publishing Group; 2013;109:1172–80.

McWilliam A, Lutter R, Nardinelli C. Healthcare impact of personalized medicine using genetic testing: An exploratory analysis for warfarin. Futur. Med. 2008;5:279–84. 133.

Meads C, Round J, Tubeuf S, Moore D, Pennant M, Bayliss S. Cetuximab for the first-line treatment of metastatic colorectal cancer. Health Technol. Assess. 2010;14 Suppl 1:1–8. 134.

Meckley LM, Gudgeon JM, Anderson JL, Williams MS, Veenstra DL. A policy model to evaluate the benefits, risks and costs of warfarin pharmacogenomic testing. Pharmacoeconomics. 2010;28:61–74. 135.

Medical Advisory Secretariat. Gene Expression Profiling for Guiding Adjuvant Chemotherapy Decisions in Women with Early Breast Cancer: An Evidence-Based and Economic Analysis [Internet]. Ont. Health Technol. Assess. Ser. 2010.

Medical Advisory Secretariat. KRAS Testing for Anti-EGFR Therapy in Advanced Colorectal Cancer: An Evidence-Based and Economic Analysis. [Internet]. Ont. Health Technol. Assess. Ser. 2010.

Medical Advisory Secretariat. Epidermal Growth Factor Receptor Mutation (EGFR) Testing for Prediction of Response to EGFR-Targeting Tyrosine Kinase Inhibitor (TKI) Drugs in Patients with Advanced Non-Small-Cell Lung Cancer: An Evidence-Based Analysis [Internet]. Ont Heal. Technol Assess Ser. 2010.

Millar JA, Millward MJ. Cost Effectiveness of Trastuzumab in the Adjuvant Treatment of Early A Lifetime Model. 2007;25:429–42. 139.

Mittmann N, Verma S, Koo M, Alloul K, Trudeau M. Cost effectiveness of TAC versus FAC in adjuvant treatment of node-positive breast cancer. Curr. Oncol. 2010;17:7–16. 140.

Mittmann N, Au H-J, Tu D, O’Callaghan CJ, Isogai PK, Karapetis CS, et al. Prospective cost-effectiveness analysis of cetuximab in metastatic colorectal cancer: evaluation of National Cancer Institute of Canada Clinical Trials Group CO.17 trial. J. Natl. Cancer Inst. 2009;101:1182–92. 141.

Muntinghe FLH, Vegter S, Verduijn M, Boeschoten EW, Dekker FW, Navis G, et al. Using a genetic, observational study as a strategy to estimate the potential cost-effectiveness of pharmacological CCR5 blockade in dialysis patients. Pharmacogenet. Genomics [Internet]. United States; 2011;21:417–25.

National institute for Health and Clinical Excellence. Trastuzumab for the adjuvant treatment of early-stage HER2-positive breast cancer. 2006. 143.

Neyt M, Huybrechts M, Hulstaert F, Vrijens F, Ramaekers D. Trastuzumab in early stage breast cancer: A cost-effectiveness analysis for Belgium. Health Policy (New. York). 2008;87:146–59. 144.

Nherera L, Marks D, Minhas R, Thorogood M, Humphries SE. Probabilistic cost-effectiveness analysis of cascade screening for familial hypercholesterolaemia using alternative diagnostic and identification strategies. Heart [Internet]. 2011;97:1175–81.

Nieves Calatrava D, Calle-Martín ÓD La, Iribarren-Loyarte JA, Rivero-Román A, García-Bujalance L, Pérez-Escolano I, et al. Cost-effectiveness analysis of HLA-B*5701 typing in the prevention of hypersensitivity to abacavir in HIV patients in Spain. Enferm. Infecc. Microbiol. Clin. 2010;28:590–5. 146.

Norum J, Olsen J a, Wist E a, Lønning PE. Trastuzumab in adjuvant breast cancer therapy. A model based cost-effectiveness analysis. Acta Oncol. [Internet]. 2007;46:153–64.

Obradovic M, Mrhar A, Kos M. Cost–effectiveness of UGT1A1 genotyping in second-line, high-dose, once every 3 weeks irinotecan monotherapy treatment of colorectal cancer. Futur. Med. 2008;9:539–49. 148.

Olgiati P, Bajo E, Bigelli M, De Ronchi D, Serretti A. Should pharmacogenetics be incorporated in major depression treatment? Economic evaluation in high- and middle-income European countries. Prog. Neuro-Psychopharmacology Biol. Psychiatry [Internet]. Elsevier Inc.; 2012;36:147–54.

Oppong R, Jit M, Smith RD, Butler CC, Melbye H, Molstad S, et al. Cost-effectiveness of point-of-care C-reactive protein testing to inform antibiotic prescribing decisions. Br. J. Gen. Pract. [Internet]. England; 2013;63:e465-71.

Panattoni L, Brown PM, Te Ao B, Webster M, Gladding P. The cost effectiveness of genetic testing for CYP2C19 variants to guide thienopyridine treatment in patients with acute coronary syndromes: a New Zealand evaluation. Pharmacoeconomics [Internet]. New Zealand; 2012;30:1067–84.

Park DJ, Kang JH, Lee JW, Lee KE, Wen L, Kim TJ, et al. Cost-effectiveness analysis of HLA-B5801 genotyping in the treatment of gout patients with chronic renal insufficiency in Korea. Arthritis Care Res. 2015;67:280–7. 152.

Parthan A, Leahy KJ, O’Sullivan AK, Iakoubova OA, Bare LA, Devlin JJ, et al. Cost effectiveness of targeted high-dose atorvastatin therapy following genotype testing in patients with acute coronary syndrome. Pharmacoeconomics [Internet]. New Zealand; 2013;31:519–31.

Patrick AR, Avorn J, Choudhry NK. Cost-effectiveness of genotype-guided warfarin dosing for patients with atrial fibrillation. Circ. Cardiovasc. Qual. Outcomes. 2009;2:429–36. 154.

Paulden M, Franek J, Pham B, Bedard PL, Trudeau M, Krahn M. Cost-effectiveness of the 21-gene assay for guiding adjuvant chemotherapy decisions in early breast cancer. Value Heal. [Internet]. Elsevier; 2013;16:729–39.

Paz-Ares L, García Del Muro X, Grande E, González P, Brosa M, Díaz S. Cost-effectiveness analysis of sunitinib in patients with metastatic and/or unresectable gastrointestinal stroma tumours (GIST) after progression or intolerance with imatinib. Clin. Transl. Oncol. 2008;10:831–9. 156.

Perez-Ellis C, Goncalves A, Jacquemier J, Marty M, Girre V, Roché H, et al. Cost-Effectiveness Analysis of Trastuzumab (Herceptin) in HER2-Overexpressed Metastatic Breast Cancer. Am. J. Clin. Oncol. [Internet]. 2009 [cited 2017 Jul 26];32:492–8.

Perlis RH, Patrick A, Smoller JW, Wang S. When is Pharmacogenetic Testing for Antidepressant Response Ready for the Clinic? A Cost-effectiveness Analysis Based on Data from the STAR*D Study. Neuropsychopharmacology. 2012;34:2227–36. 158.

Petta S, Cabibbo G, Enea M, Macaluso FS, Plaia A, Bruno R, et al. Personalized cost-effectiveness of boceprevir-based triple therapy for untreated patients with genotype 1 chronic hepatitis C. Dig. Liver Dis. [Internet]. Editrice Gastroenterologica Italiana; 2014;46:936–42.

Pichereau S, Le Louarn A, Lecomte T, Blasco H, Le Guellec C, Bourgoin H. Cost-effectiveness of UGT1A1*28 genotyping in preventing severe neutropenia following FOLFIRI therapy in colorectal cancer. J. Pharm. Pharm. Sci. 2010;13:615–25. 160.

Pink J, Pirmohamed M, Lane S, Hughes DA. Cost-effectiveness of pharmacogenetics-guided warfarin therapy vs. alternative anticoagulation in atrial fibrillation. Clin Pharmacol Ther. 2014;95:199–207. 161.

Plumpton CO, Yip VLM, Alfirevic A, Marson AG, Pirmohamed M, Hughes DA. Cost-effectiveness of screening for HLA-A*31:01 prior to initiation of carbamazepine in epilepsy. Epilepsia [Internet]. United States; 2015;56:556–63.

Poncet B, Bachelot T, Colin C, Ganne C, Jaisson-Hot I, Orfeuvre H, et al. Use of the Monoclonal Antibody Anti-HER2 Trastuzumab in the Treatment of Metastatic Breast Cancer. Am. J. Clin. Oncol. [Internet]. 2008 [cited 2017 Jul 26];31:363–8.

Purmonen TT, Pänkäläinen E, Turunen JHO, Asseburg C, Martikainen JA. Short-course adjuvant trastuzumab therapy in early stage breast cancer in Finland: cost-effectiveness and value of information analysis based on the 5-year follow-up results of the FinHer Trial. Acta Oncol. [Internet]. 2011;50:344–52.

Rattanavipapong W, Koopitakkajorn T, Praditsitthikorn N, Mahasirimongkol S, Teerawattananon Y. Economic evaluation of HLA-B*15:02 screening for carbamazepine- induced severe adverse drug reactions in Thailand. Epilepsia. 2013;54:1628–38. 165.

Reed SD, Scales CD, Stewart SB, Sun J, Moul JW, Schulman KA, et al. Effects of family history and genetic polymorphism on the cost-effectiveness of chemoprevention with finasteride for prostate cancer. J. Urol. 2011;185:841–7. 166.

Reed SD, Dinan MA, Schulman KA, Lyman GH. Cost-effectiveness of the 21-gene recurrence score assay in the context of multifactorial decision making to guide chemotherapy for early-stage breast cancer. Genet. Med. [Internet]. 2013;15:203–11.

Reese ES, Mullins CD, Beitelshees AL, Onukwugha E. Cost-Effectiveness of Cytochrome P450 2C19 Genotype Screening for Selection of Antiplatelet Therapy with Clopidogrel or Prasugrel. Pharmacotherapy. 2012;32:323–32. 168.

Refaat T, Choi M, Gaber G, Kiel K, Mehta M, Gradishar W, et al. Markov Model and Cost-Effectiveness Analysis of Bevacizumab in HER2-Negative Metastatic Breast Cancer. Am. J. Clin. Oncol. [Internet]. 2014;37:480–5.

Retèl VP, Joore MA, Drukker CA, Bueno-De-Mesquita JM, Knauer M, Van Tinteren H, et al. Prospective cost-effectiveness analysis of genomic profiling in breast cancer. Eur. J. Cancer. 2013;49:3773–9. 170.

Retèl VP, Joore M a., Knauer M, Linn SC, Hauptmann M, Harten WH Van. Cost-effectiveness of the 70-gene signature versus St. Gallen guidelines and Adjuvant Online for early breast cancer. Eur. J. Cancer. 2010;46:1382–91. 171.

Retèl VP, Joore M a, Linn SC, Rutgers EJT, van Harten WH. Scenario drafting to anticipate future developments in technology assessment. BMC Res. Notes [Internet]. 2012;5:442.

Retèl VP, Joore M a, van Harten WH. Head-to-head comparison of the 70-gene signature versus the 21-gene assay: cost-effectiveness and the effect of compliance. Breast Cancer Res. Treat. [Internet]. 2012;131:627–36.

Risebrough NA, Verma S, Trudeau M, Mittmann N. Cost-effectiveness of switching to exemestane versus continued tamoxifen as adjuvant therapy for postmenopausal women with primary breast cancer. Cancer. 2007;110:499–508. 174.

Romanus D, Cardarella S, Cutler D, Landrum MB, Lindeman NI, Gazelle GS. Cost-effectiveness of multiplexed predictive biomarker screening in non-small-cell lung cancer. J. Thorac. Oncol. [Internet]. United States; 2015;10:586–94.

Rubinstein WS, Jiang H, Dellefave L, Rademaker AW. Cost-effectiveness of population-based BRCA1/2 testing and ovarian cancer prevention for Ashkenazi Jews: A call for dialogue. Genet. Med. [Internet]. 2009 [cited 2017 Jul 26];11:629–39.

Sanon M, Taylor DC, Parthan A, Coombs J, Paolantonio M, Sasane M. Cost-effectiveness of 3-years of adjuvant imatinib in gastrointestinal stomal tumors (GIST) in the United States. J. Med. Econ. 2013;16:150–9. 177.

Saokaew S, Tassaneeyakul W, Maenthaisong R, Chaiyakunapruk N. Cost-effectiveness analysis of HLA-B*5801 testing in preventing allopurinol-induced SJS/TEN in Thai population. PLoS One. 2014;9:1–9. 178.

Schackman BR, Scott CA, Walensky RP, Losina E, Freedberg KA, Sax PE. The cost-effectiveness of HLA-B*5701 genetic screening to guide initial antiretroviral therapy for HIV. AIDS. 2008;22. 179.

Segui MA, Crespo C, Cortes J, Lluch A, Brosa M, Becerra V, et al. Genomic profile of breast cancer: cost-effectiveness analysis from the Spanish National Healthcare System perspective. Expert Rev. Pharmacoecon. Outcomes Res. [Internet]. England; 2014;14:889–99.

Sendi P, Günthard HF, Simcock M, Ledergerber B, Schüpbach J, Battegay M. Cost-effectiveness of genotypic antiretroviral resistance testing in HIV-infected patients with treatment failure. PLoS One. 2007;2. 181.

Serretti A, Olgiati P, Bajo E, Bigelli M, De Ronchi D. A model to incorporate genetic testing (5-HTTLPR) in pharmacological treatment of major depressive disorders. World J. Biol. Psychiatry [Internet]. England; 2011;12:501–15.

Sher DJ, Tishler RB, Annino D, Punglia RS. Cost-effectiveness of CT and PET-CT for determining the need for adjuvant neck dissection in locally advanced head and neck cancer. Ann. Oncol. 2009;21:1072–7. 183.

Shiffman D, Slawsky Katherine, Fusfeld L, Devlin JJ, Goss TF. Cost-Effectiveness Model of Use of Genetic Testing as an Aid in Assessing the Likely Benefit of Aspirin Therapy for Primary Prevention of Cardiovascular Disease. Clin. Ther. [Internet]. Elsevier; 2012 [cited 2017 Sep 19];34:1387–94.

Shih V, Chan A, Xie F, Ko Y. Economic Evaluation of Anastrozole Versus Tamoxifen for Early Stage Breast Cancer in Singapore. Value Heal. Reg. Issues. 2012;1:46–53. 185.

Shiroiwa T, Motoo Y, Tsutani K. Pcn16 Cost-Effectiveness Analysis of K-Ras Testing and Cetuximab for Metastatic Colorectal Cancer in Japan. Value Heal. [Internet]. 2010;13:A513.

Shiroiwa T, Fukuda T, Shimozuma K, Ohashi Y, Tsutani K. The model-based cost-effectiveness analysis of 1-year adjuvant trastuzumab treatment: Based on 2-year follow-up HERA trial data. Breast Cancer Res. Treat. 2008;109:559–66. 187.

Siebert U, Sroczynski G, Aidelsburger P, Rossol S, Wasem J, Manns MP, et al. Clinical effectiveness and cost effectiveness of tailoring chronic hepatitis c treatment with peginterferon alpha-2b plus ribavirin to hcv genotype and early viral response: A Decision analysis based on german guidelines. Pharmacoeconomics. 2009;27:341–54. 188.

Skedgel C, Rayson D, Dewar R, Younis T. Cost-utility of adjuvant hormone therapies for breast cancer in post-menopausal women: Sequential tamoxifen-exemestane and upfront anastrozole. Breast Cancer Res. Treat. 2007;101:325–33. 189.

Skedgel C, Rayson D, Dewar R, Younis T. Cost-utility of adjuvant hormone therapies with aromatase inhibitors in post-menopausal women with breast cancer: Upfront anastrozole, sequential tamoxifen-exemestane and extended tamoxifen-letrozole. Breast. 2007;16:252–61. 190.

Skedgel C, Rayson D, Younis T. The cost-utility of sequential adjuvant trastuzumab in women with Her2/Neu-Positive Breast Cancer: An analysis based on updated results from the HERA trial. Value Heal. [Internet]. International Society for Pharmacoeconomics and Outcomes Research (ISPOR); 2009;12:641–8.

Smith KJ, Monsef BS, Ragni M V. Should female relatives of factor V Leiden carriers be screened prior to oral contraceptive use? A cost-effectiveness analysis. Thromb. Haemost. 2008;100:447–52. 192.

Sorich MJ, Horowitz JD, Sorich W, Wiese MD, Pekarsky B, Karnon JD. Cost-effectiveness of using CYP2C19 genotype to guide selection of clopidogrel or ticagrelor in Australia. Pharmacogenomics [Internet]. 2013;14:2013–21.

Sullivan SD, Garrison LP, Rinde H, Kolberg J, Moler EJ. Cost-effectiveness of risk stratification for preventing type 2 diabetes using a multi-marker diabetes risk score. J. Med. Econ. [Internet]. 2011;14:609–16.

Suter LG, Fraenkel L, Braithwaite S. Cost-effectiveness of Adding Magnetic Resonance Imaging to Rheumatoid Arthritis Management. Arch. Intern. Med. 2011;171:657–67. 195.

Thompson AJ, Newman WG, Elliott RA, Roberts SA, Tricker K, Payne K. The cost-effectiveness of a pharmacogenetic test: a trial-based evaluation of TPMT genotyping for azathioprine. Value Health [Internet]. United States; 2014;17:22–33.

Thompson D, Taylor DCA, Montoya EL, Winer EP, Jones SE, Weinstein MC. Cost-effectiveness of switching to exemestane after 2 to 3 years of therapy with tamoxifen in postmenopausal women with early-stage breast cancer. Value Heal. [Internet]. International Society for Pharmacoeconomics and Outcomes Research (ISPOR); 2007;10:367–76.

Thongprasert S, Tinmanee S, Permsuwan U. Cost-utility and budget impact analyses of gefitinib in second-line treatment for advanced non-small cell lung cancer from Thai payer perspective. Asia. Pac. J. Clin. Oncol. 2011;8:53–61. 198.

Tiamkao S, Jitpimolmard J, Sawanyawisuth K, Jitpimolmard S. Cost minimization of HLA-B*1502 screening before prescribing carbamazepine in Thailand. Int. J. Clin. Pharm. 2013;35:608–12. 199.

Tsoi DT, Inoue M, Kelly CM, Verma S, Pritchard KI. Cost-Effectiveness Analysis of Recurrence Score-Guided Treatment Using a 21-Gene Assay in Early Breast Cancer. Oncologist [Internet]. 2010;15:457–65.

van Loon J, Grutters JPC, Wanders R, Boersma L, Dingemans AMC, Bootsma G, et al. 18FDG-PET-CT in the follow-up of non-small cell lung cancer patients after radical radiotherapy with or without chemotherapy: An economic evaluation. Eur. J. Cancer [Internet]. Elsevier Ltd; 2010;46:110–9.

Van Vlaenderen I, Canon JL, Cocquyt V, Jerusalem G, Machiels JP, Neven P, et al. Trastuzumab Treatment of Early Stage Breast Cancer Is Cost-Effective From the Perspective of the Belgian Health Care Authorities. Acta Clin. Belg. [Internet]. 2009;64:100–12.

Vanderlaan BF, Broder MS, Chang EY, Oratz R, Bentley TGK. Cost-effectiveness of 21-gene assay in node-positive, early-stage breast cancer. Am. J. Manag. Care. 2011;17:455–64. 203.

Veenstra DL, Harris J, Gibson RL, Rosenfeld M, Burke W, Watts C. Pharmacogenomic testing to prevent aminoglycoside-induced hearing loss in cystic fibrosis patients: potential impact on clinical, patient, and economic outcomes. Genet. Med. 2007;9:695–704. 204.

Vegter S, Perna A, Hiddema W, Ruggenenti P, Remuzzi G, Navis G, et al. Cost-effectiveness of ACE inhibitor therapy to prevent dialysis in nondiabetic nephropathy: influence of the ACE insertion/deletion polymorphism. Pharmacogenet Genomics [Internet]. 2009;19:695–703.

Verhoef TI, Redekop WK, Veenstra DL, Thariani R, Beltman PA, van Schie RM, et al. Cost-effectiveness of pharmacogenetic-guided dosing of phenprocoumon in atrial fibrillation. Pharmacogenomics [Internet]. 2013;14:869–83.

Verry H, Lord SJ, Martin A, Gill G, Lee CK, Howard K, et al. Effectiveness and cost-effectiveness of sentinel lymph node biopsy compared with axillary node dissection in patients with early-stage breast cancer: A decision model analysis. Br. J. Cancer [Internet]. Nature Publishing Group; 2012;106:1045–52.

Vijayaraghavan A, Efrusy MB, Göke B, Kirchner T, Santas CC, Goldberg RM. Cost-effectiveness of KRAS testing in metastatic colorectal cancer patients in the United States and Germany. Int. J. Cancer [Internet]. 2012;131:438–45.

Walleser S, Ray J, Bischoff H, Vergnenègre A, Rosery H, Chouaid C, et al. Maintenance erlotinib in advanced nonsmall cell lung cancer: Cost-effectiveness in EGFR wild-type across Europe. Clin. Outcomes Res. 2012;4:269–75. 209.

Wang S, Peng L, Li J, Zeng X, Ouyang L, Tan C, et al. A Trial-Based Cost-Effectiveness Analysis of Erlotinib Alone versus Platinum-Based Doublet Chemotherapy as First-Line Therapy for Eastern Asian Nonsquamous Non–Small-Cell Lung Cancer. Minna JD, editor. PLoS One [Internet]. Public Library of Science; 2013 [cited 2017 Jan 24];8:e55917.

Welton NJ, Johnstone EC, David SP, Munafò MR. A cost-effectiveness analysis of genetic testing of the DRD2 Taq1A polymorphism to aid treatment choice for smoking cessation. Nicotine Tob. Res. [Internet]. 2008;10:231–40.

Wolf E, Blankenburg M, Bogner JR, Becker W, Gorriahn D, Mueller MC, et al. Cost impact of prospective HLA-B*5701-screening prior to abacavir/lamivudine fixed dose combination use in Germany. Eur. J. Med. Res. [Internet]. England; 2010;15:145–51.

Wolowacz SE, Cameron DA, Tate HC, Bagust A. Docetaxel in combination with doxorubicin and cyclophosphamide as adjuvant treatment for early node-positive breast cancer: A cost-effectiveness and cost-utility analysis. J. Clin. Oncol. 2008;26:925–33. 213.

Woods B, Veenstra D, Hawkins N. Prioritizing pharmacogenetic research: a value of information analysis of CYP2D6 testing to guide breast cancer treatment. Value Health [Internet]. United States; 2011;14:989–1001.

Yamauchi H, Nakagawa C, Yamashige S, Takei H, Yagata H, Yoshida A, et al. Societal cost-effectiveness analysis of the 21-gene assay in estrogen-receptor-positive, lymph-node-negative early-stage breast cancer in Japan. BMC Health Serv. Res. [Internet]. 2014;14:372.

Yang M, Rajan S, Issa AM. Cost effectiveness of gene expression profiling for early stage breast cancer: A decision-analytic model. Cancer. 2012;118:5163–70. 216.

Yazdanpanah Y, Vray M, Meynard J, Losina E, Weinstein MC, Morand-Joubert L, et al. The long-term benefits of genotypic resistance testing in patients with extensive prior antiretroviral therapy: a model-based approach. HIV Med. [Internet]. 2007;8:439–50.

Yen RF, Yen MF, Hong RL, Tzen KY, Chien CR, Chen THH. The Cost-utility Analysis of 18-Fluoro-2-Deoxyglucose Positron Emission Tomography in the Diagnosis of Recurrent Nasopharyngeal Carcinoma. Acad. Radiol. [Internet]. AUR; 2009;16:54–60.

You JH, Tsui KK, Wong RS, Cheng G. Potential Clinical and Economic Outcomes of CYP2C9 and VKORC1 Genotype-Guided Dosing in Patients Starting Warfarin Therapy. 2009 [cited 2017 Jul 26];

You JHS. Pharmacogenetic-guided selection of warfarin versus novel oral anticoagulants for stroke prevention in patients with atrial fibrillation: A cost-effectiveness analysis. Pharmacogenet. Genomics. 2014;24:6–14. 220.

You JHS, Tsui KKN, Wong RSM, Cheng G. Cost-effectiveness of Dabigatran versus genotype-guided management of Warfarin therapy for stroke prevention in patients with Atrial fibrillation. PLoS One. 2012;7:1–9. 221.

Zanocco K, Heller M, Elaraj D, Sturgeon C. Cost effectiveness of intraoperative pathology examination during diagnostic hemithyroidectomy for unilateral follicular thyroid neoplasms. J. Am. Coll. Surg. [Internet]. Elsevier Inc; 2013;217:702–10.

Zhu J, Li T, Wang X, Ye M, Cai J, Xu Y, et al. Gene-guided gefitinib switch maintenance therapy for patients with advanced EGFR mutation-positive non-small cell lung cancer: an economic analysis. BMC Cancer [Internet]. 2013;13:39.

Table 1: Location of Economic Evaluations of Precision Medicines (2007-2017)

| **Country** | **Number of Studies^1^** | **Proportion of Studies (%)** |
| --- | --- | --- |
| USA | 68 | 30 |
| Canada | 29 | 13 |
| UK | 27 | 12 |
| The Netherlands | 11 | 5 |
| Japan | 10 | 4 |
| Germany | 10 | 4 |
| Switzerland | 7 | 3 |
| France | 7 | 3 |
| Italy | 7 | 3 |
| China | 5 | 2 |
| Australia | 5 | 2 |
| Singapore | 4 | 2 |
| Sweden | 4 | 2 |
| Thailand | 4 | 2 |
| Spain | 3 | 1 |
| South Korea | 3 | 1 |
| Europe | 3 | 1 |
| Belgium | 3 | 1 |
| Brazil | 2 | 1 |
| Norway | 2 | 1 |
| Taiwan | 2 | 1 |
| Mexico | 2 | 1 |
| Iran | 1 | 0 |
| Greece | 1 | 0 |
| Colombia | 1 | 0 |
| Ireland | 1 | 0 |
| Israel | 1 | 0 |
| New Zealand | 1 | 0 |
| Finland | 1 | 0 |
| Saudi Arabia | 1 | 0 |
| South Africa | 1 | 0 |

^1^ This column sums to more than 222 as some studies were conducted in multiple countries

Table 5: Focus of Economic Evaluations of Precision Medicine(2007-2017)

| **Condition** | **Number of Studies**  **(Total n=222)** |
| --- | --- |
| Breast Cancer | 104 |
| Cardiovascular Conditions^a^ | 22 |
| Lung Cancer | 18 |
| Colorectal Cancer | 15 |
| HIV | 12 |
| Infectious Disease ^b^ | 7 |
| Gastrointestinal Cancer | 5 |
| Epilepsy | 4 |
| Smoking Cessation | 4 |
| Rheumatoid Arthritis | 3 |
| Depression | 3 |
| Thyroid Nodules | 3 |
| Gout | 2 |
| Renal Disorders | 2 |
| Diabetes | 2 |
| Ovarian cancer | 2 |
| Endometrial Cancer | 1 |
| Contraception | 1 |
| Autoimmune Disease | 1 |
| Cystic Fibrosis | 1 |
| Non-diabetic Neuropathy | 1 |
| Pediatric Cancers | 1 |
| Leukemia | 1 |
| A range of cancers | 1 |
| Pulmonary Fibrosis | 1 |
| Familial Hypercholosterolemia | 1 |
| Mild Cognitive Impairment | 1 |
| Abdominal lesions | 1 |
| Malignant Melanoma | 1 |
| Prostate cancer | 1 |
| Head/Neck Cancers | 1 |
| Nasophayrngal Cancers | 1 |

^a^ including acute myocardial infarction, stroke prevention and acute coronary syndrome

^b^ including Hepatitis C
