# Supplementary material for: Accounting for Capacity Constraints in Economic Evaluations of Precision Medicine: A Systematic Review
Source: Pharmacoeconomics. 2019 May 13;37(8):1011–27. doi: 10.1007/s40273-019-00801-9 (PMC6597608; doi:10.1007/s40273-019-00801-9)
Supplement: Supplementary file 4 — Supplementary material 4 (DOCX 33 kb) [file 40273_2019_801_MOESM4_ESM.docx]

PharmacoEconomics. Accounting for Capacity Constraints in Economic Evaluations of Precision Medicine: a Systematic Review. Stuart J Wright, William Newman, Katherine Payne

Correspondence to Stuart J Wright, Manchester Centre for Health Economics, Division of Population Health, Health Services Research & Primary Care, The University of Manchester, Oxford Road, Manchester, M13 9PL, stuart.wright-2@manchester.ac.uk, 01613067970

Supplementary Appendix 4: Summary of identified studies which include capacity constraints

| **Author (Year)**  **Country** | **Disease Area** | **Intervention and Comparator** | **How did the study discuss capacity?** | **Did the study quantify the impact of capacity constraints in the analysis? If so, what methods were used?** |
| --- | --- | --- | --- | --- |
| Barone et al., (2014) (1)  Italy | Colorectal Cancer | Intervention: KRAS testing to guide treatment with cetuximab and bevacizumab  Comparator: Standard chemotherapy | Used data that suggested 75% of experts thought that test turnaround time affects treatment choice. Turnaround longer than 15 days could limit therapy choice and 10 days is considered optimal. However, 25% of participants in a survey recording turnaround time stated a delay of over 15 days. | No |
| Breijer et al., (2012) (2)  The Netherlands | Endometrial Cancer | Intervention: Three transvaginal sonography-based diagnostic strategies for endometrial cancer for women with post-menopausal bleeding.  Comparator: Diagnosis based on patient history only | Recommend the use of patient characteristics to target diagnostics more effectively  Highlighted the need to make the diagnostic stratifying algorithm easy to access and use for GPs due to small expected gains per patient | No |
| Chen and Dowdy (2014) (3)  USA | HIV | Intervention: Risk calculators to guide HIV pre-exposure prophylaxis use in men who have sex with men  Comparator: No pre-exposure prophylaxis | Economic evidence may be useful in guiding the implementation of interventions at a local and state level | No |
| Collinson et al., (2013) (4)  UK | Acute Myocardial Infarction | Intervention: Blood based troponin assays and cardiac biomarkers for detecting AMI    Comparator: Hospital admission and 10 to 12 hour troponin measurement | Made suggestions in a general observations section that test performance for new biomarkers should be adequate and that the marker should be measurable in a routine laboratory. Went on to say that measurement should be precise and accurate and there should be a rapid turnaround time. Furthermore suggesting that ideally testing should be available with current lab resources and not require additional apparatus. | No |
| Contreras-Hernandez et al., (2008) (5)  Mexico | Gastrointestinal Cancer | Intervention: Second-line treatment with imatinib or sunitinib in patients with advanced gastrointestinal stromal tumours  Comparator: Palliative care | Stated the need to take account of the fact that budgets of medical payers are constrained | No |
| Delea et al., (2012) (6)  UK | Breast Cancer | Intervention: Lapatinib and capecitabine for women with HER2 positive metastatic breast cancer who have previously received trastuzumab  Comparator: Capecitabine monotherapy | Included a figure for drug wastage. Mentioned that the drug wastage parameter they included was similar to the value found in an Italian study prior to the implementation of a strategy to reduce waste. | Yes: an average drug wastage number was used in the analysis. This number was set to zero in sensitivity analysis, reducing total costs. This suggests cost-effectiveness of intervention depends on implementation. |
| Delea et al., (2013) (7)  UK | Breast Cancer | Intervention: Lapatinib and letrozole for post-menopaulsal women with hormone receptor and HER2 positive metastatic breast cancer  Comparator: Trastuzumab and anastrozole or trastuzumab alone or letrozole alone | The authors noted that a lower rate of drug wastage after full programme implementation was observed | Yes: an average drug wastage number was used in the analysis. This number was set to zero in sensitivity analysis, reducing total costs. This suggests cost-effectiveness of intervention depends on implementation. |
| Dionne et al., (2012) (8)  Canada | Paediatric Cancer | Intervention: Genetic test to predict susceptibility to adverse events of cisplatin based chemotherapy  Comparator: No test. | Suggested that test costs could change with higher utilisation and technological advances | No |
| Djalalov et al., (2014) (9)  Canada | Lung Cancer | Intervention: EML4-ALK Fusion testing and  first-line crizotinib treatment for patients with advanced  ALK-positive non–small-cell lung cancer  Comparator: Platinum doublet chemotherapy | Suggested there were challenges to implementing molecular testing with FISH for ALK fusions: large number of NSCLC patients by low level of the mutation, testing; complexity of test; testing cost; need for sufficient tissue sample. | Yes: the decision tree includes a branch for to represent whether there is an adequate tissue sample and if not allows for a second biopsy to be taken. It is not clear if these probabilities were varied in sensitivity analysis but the cost of re-biopsy was allowed to vary. |
| Essers et al., (2010) (10)  The Netherlands | Breast Cancer | Intervention: Trastuzumab for the adjuvant treatment of HER2 positive early breast cancer  Comparator: Observation and trastuzumab if progression to metastatic disease | Suggested that improving the transferability of economic evaluations may improve speed of reimbursement of new pharmaceuticals | No |
| Garrison and Veenstra (2009) (11)  USA | Breast Cancer | Intervention: Trastuzumab for various stages of HER2 positive breast cancer  Comparator: Unclear | Suggested that increasing the number of indications for a drug and, therefore, the number of eligible patients will change relative cost-effectiveness over time. However, the analysis still assumed that all patients immediately have access to the treatment when their indication was approved | Yes: the analysis quantified dynamic cost-effectiveness with changing patient population and implied that limited approved indications for a drug may inhibit potential cost-effectiveness |
| Garrison et al., (2013) (12)  USA | Breast Cancer | Intervention: Retesting IHC tested HER2 negative patients for HER2 with FISH to reduce false-negative results  Comparator: Current testing algorithm (no retesting) | Suggested several factors that will affect the implementation of expanded reflex testing. Many labs only have the capacity to perform IHC and not FISH. Improvements in capacity and investment in equipment may be needed. However the requirements on increased capacity and equipment would be even greater if full initial HER2 testing were implemented. Uncertainty about whether payers would accept an expansion to reflex testing which would see the proportion of patients receiving FISH testing rise from 26.6% to 86.9%. Newly approved testing techniques such as silver in situ hybridization may reduce the analytic burden of reflex testing. | No |
| Hornberger et al., (2011) (13)  USA | Breast Cancer | Intervention: 21-gene assay to guide treatment of patients with estrogen receptor positive, lymph node negative early stage breast cancer  Comparator: Unclear | Suggested the relative cost-effectiveness of the assay may change with the volume of testing in clinical practice as a result of learning effects by clinicians using the assay | No |
| Ito et al., (2013) (14)  USA | Breast Cancer | Intervention: Eliminating co-payments made by Medicare beneficiaries for aromatase inhibitors for patients with hormone-receptor positive early breast cancer  Comparator: Existence of co-payments by Medicare beneficiaries for aromatase inhibitors for patients with hormone-receptor positive early breast cancer | Suggested that the drugs may be underused due to cost constraints but unclear whether from what was written whether this was from an organisational or patient perspective | No |
| Klang et al., (2010) (15)  Israel | Breast Cancer | Intervention: Oncotype DX use in women with estrogen receptor positive, lymph-node negative early stage breast cancer  Comparator: “Traditional prognostic pathways” | Suggested that clinicians would need to learn about the technology and how to interpret tests | No |
| Kondo et al., (2008) (16)  Japan | Breast Cancer | Intervention: 21-gene reverse transcriptase-polymerase chain reaction assay in guiding treatment for lymph-node-negative, estrogen-receptor-positive, early-stage breast cancer  Comparator: National Comprehensive Cancer Network (NCCN) guideline/St Gallen recommendation-  guided treatment | Suggested the need to consider the financial implications of needing an additional 12,000 tests a year and the need to do a budget impact analysis | Yes: used budget impact analysis to determine total cost implication |
| Lala et al., (2013) (17)  USA | Acute Coronary Syndrome | Intervention: CYP2C19 gene testing to guide antiplatelet therapy in patients with acute coronary syndrome undergoing percutaneous coronary intervention  Comparator: Two no-testing strategies | Suggested that the time between performing the test and receiving test results is likely to shorten over time and with the advent of new technology making point of care testing more feasible | No |
| Lee et al., (2010) (18)  South Korea | Breast Cancer | Intervention: Adjuvant hormonal treatments for women with postmenopausal hormone-receptor positive early breast cancer  Comparator: Tamoxifen | Suggested that the availability of treatment might be limited given the high cost of the drug and increasing healthcare budget constraints. | No |
| Lidgren et al., (2008b) (19)  Sweden | Breast Cancer | Intervention: HER2 testing and trastuzumab in combination with chemotherapy for metastatic breast cancer  Comparator: Chemotherapy alone | Concluded that even if drug is a cost-effective use of resources that some clinicians may not prescribe it due to budget constraints. This would lead to inequitable access to treatment for patients | No |
| Lorenzana et al., (2012) (20)  South Africa | HIV | Intervention: Genotype assay for selection of  third-line antiretroviral therapy (ART) in resource-limited settings, as per the planned international  A5288 trial  Comparator: No assay and second-line ART and no assay and third-line ART | Suggested that tests costs are often cited as a barrier to implementation. The cost of the genotype test was found to have little impact on cost-effectiveness but making the genotype test available may require additional investment in physical and human resources. These additional resources were excluded from the analysis. Investment in these resources may need to be significant to produce an acceptable turnaround time of less than two-months. | Yes – test cost was apparently varied in sensitivity analysis with suggestions that higher test cost could represent cost when investment is accounted for. No impact on cost-effectiveness found. |
| Machanda et al., (2015)(21)  UK and USA | Breast Cancer | Intervention: Population based BRCA testing with  varying Ashkenazi Jewish ancestry  Comparator: No BRCA testing | Suggested that to implement a population based testing strategy there will need to be wide-scale dissemination of information and knowledge involving working with stake holders and health professionals. Other issues may be faced with “health system delivery, referral and management pathways, logistics, and control, which can vary across different models of care in different countries” | No |
| McCowan et al., (2013) (22)  UK | Breast Cancer | Intervention: High adherence to tamoxifen in women with breast cancer  Comparator: Low adherence (<80%) | The issues raised in the paper focussed on adherence and as the authors state “besides patient-related factors, the characteristics of the disease and its treatment, the attributes of  the health-care system and service delivery may also influence adherence”. Lower adherence was linked to shorter time to cancer recurrence and higher health service costs later on. Interventions which improve adherence should improve outcomes and may be highly cost-effective | Yes – evaluation conducted across sub-groups of patients with under or over 80% adherence. Low adherence associated with expected loss of 1.12 discounted QALYs and increase of £5,970 in medical costs.  Methods could be extrapolated to compare cost-effectiveness of high patient access to treatments. |
| Medical Advisory Secretariat (2010) (23)  Canada | Lung Cancer | Intervention: Epidermal growth factor Receptor mutation (EGFR) testing for prediction of response to EGFR- targeting tyrosine kinase Inhibitor (TKI) drugs in patients with advanced non-small-cell lung cancer  Comparator: First-line cisplatin and gemcitabine, second-line docetaxel or pemetrexed and third-line erlotinib | Suggested there are barriers to routine FISH testing including lack of expertise in molecular techniques and lack of experience with dark-field fluorescence microscopy. IHC testing is quick and simple and uses available equipment and reagents. | No |
| Medical Advisory Secretariat (2010b) (24)  Canada | Breast Cancer | Intervention: Gene expression profiling for guiding adjuvant chemotherapy decisions in women with early estrogen or progesterone receptor positive breast cancer  Comparator: No testing | Suggested that the cost of testing meant that economic evaluation needed as a step before introducing into dynamic health systems. Also state that “clinician communication affects the usefulness of test information” for treatment decision making. Furthermore a lack of genetic literacy amongst patients may make it hard for them to fully take on information from genetic tests. | No |
| Patrick et al., (2009) (25)  USA | Stroke Prevention | Intervention: Genotype-guided (CYP2C9 and VKORC1) warfarin dosing for patients with atrial fibrillation  Comparator: No testing | Suggested that the total cost of implementing genetic testing would have significant economic consequences | No |
| Retèl et al., (2010) (26)  The Netherlands | Breast Cancer | Intervention:70-gene signature MammaPrint for guiding treatment of patients with node negative breast cancer  Comparator: St Gallen guidelines and Adjuvant online algorithm for guiding treatment of patients with node negative breast cancer | Stated that the analysis assumed perfect implementation and that the cost-effectiveness results represent the intervention being used in a perfect way. | No |
| Retèl et al., (2012) (27)  The Netherlands | Breast Cancer | Intervention: Potential scenarios for the diffusion of a 70-gene signature into clinical practice  Comparator: Retaining the St Gallan guidelines and Adjuvant! Online algorithm for decision making | The stated focus of the analysis in this paper is to consider the impact of partial implementation of a new technology. The analysis factored in a range of scenarios including barriers or removal of barriers. The scenarios evaluated were: hesitant adopters, increased user-friendliness, proof of the value of RNA-preservation, adoption in national guidelines of the test, improved reimbursement of testing, introduction of competitor tests, Oncotype dx (a rival test) is revealed to be better than the Mammaprint test, the test is made obsolete by a newer test, the test becomes available on the free market, and the better regulation of Mammaprint improves its market share versus Oncotype dx. The cost-effectiveness of Mammaprint over time and the diffusion of the intervention was explored by the researchers. | Yes – the researchers modelled the cost-effectiveness over time and diffusion of the technology. They include a range of potential scenarios and barriers which affect the diffusion of the technology |
| Romanus et al., (2015) (28)  USA | Lung Cancer | Intervention: Multiplexed testing for EGFR and ALK mutations to guide NSCLC treatment  Comparator: No testing and treatment with pemetrexed and cisplatin | Accounted for the issue that long turnaround times for tests and inadequate tissue samples may push the balance of testing decisions towards generic chemotherapy use. | Yes – included a parameter for turnaround time and inadequate tissue sample leading to re-biopsy as well as proportion of patients tested |
| Rubinstein et al., (2009) (29)  USA | Breast Cancer | Intervention: Population-based BRCA1/2 testing and ovarian cancer prevention for Ashkenazi Jews  Comparator: No testing | Suggested that improvements in other technologies such as MRI scans may cause additional costs and benefits for the intervention of interest when they are implemented. They also suggested that active implementation frameworks rather than passive uptake of guidelines may help the health system to achieve economies of scale and allow better evaluation of the provision of the intervention. | No |
| Saokaew at al., (2014) (30)  Thailand | Gout | Intervention: HLA-B5801 genotyping of gout patients with a high risk of allopurinol-induced severe cutaneous adverse reactions before treatment with allopurinol  Comparator: No genetic testing | Stated that the analysis did not account for the feasibility of implementing the proposed test or take account of the affordability of the test. They also suggested that there is a need for clinicians to have an understanding of the test and its implications. | No |
| Segui et al., (2014) (31)  Spain | Breast Cancer | Intervention: 70-gene signature to assess tumour recurrence risk and determine chemotherapy treatment  Comparator: No testing | Suggested that the technical ease of use and pricing of the assay will be important when considering the implementation of tests into clinical practice. | No |
| Thompson et al., (2014) (32)  UK | Autoimmune Diseases | Intervention: Thiopurine-methyl transferase testing prior to prescription of azathioprine to predict susceptibility to adverse drug reactions  Comparator: No testing. | Suggested that the results observed in the prospective trial were the results not adhering to test recommendations regarding the dose of azathioprine, which infers there might be imperfect implementation of the test in clinical practice. | No |
| Vanderlaan et al., (2011) (33)  USA | Breast Cancer | Intervention: 21-gene assay for women with early-stage, minimally node-positive, estrogen receptor–positive (N+(1-3)/ER+) HER2-negative breast cancer  Comparator: No assay | A sensitivity analysis included variations in utilisation rates of testing. However, as the costs and benefits of testing were not dependent on the utilisation rate, the cost-effectiveness of the intervention was not affected by changes in the utilisation rate. | Yes – sensitivity analysis included variations in utilisation rates of testing ranging from X to Y. The analysis assumed the marginal costs of the assay were linear so this analysis showed no impact on the relative cost-effectiveness of the assay |

**References**

1. Barone C, Pinto C, Normanno N, Capussotti L, Cognetti F, Falcone A, et al. KRAS early testing: Consensus initiative and cost-effectiveness evaluation for metastatic colorectal patients in an italian setting. PLoS One. 2014;9(1).

2. Breijer MC, Van Doorn HC, Clark TJ, Khan KS, Timmermans A, Mol BWJ, et al. Diagnostic strategies for endometrial cancer in women with postmenopausal bleeding: Cost-effectiveness of individualized strategies. Eur J Obstet Gynecol Reprod Biol [Internet]. Elsevier Ireland Ltd; 2012;163(1):91–6. Available from: http://dx.doi.org/10.1016/j.ejogrb.2012.03.025

3. Chen A, Dowdy DW. Clinical effectiveness and cost-effectiveness of HIV pre-exposure prophylaxis in men who have sex with men: Risk calculators for real-world decision-making. PLoS One. 2014;9(10).

4. Collinson P, Gaze D, Goodacre S, Bradburn M. RATPAC CBE (Randomised Assessment of Treatment using Panel Assay of Cardiac markers - Contemporary Biomarker Evaluation). Health Technol Assess (Rockv). 2013;17(15):1–17.

5. Contreras-Hernández I, Mould-Quevedo JF, Silva A, Salinas-Escudero G, Villasís-Keever MA, Granados-García V, et al. A pharmaco-economic analysis of second-line treatment with imatinib or sunitinib in patients with advanced gastrointestinal stromal tumours. Br J Cancer [Internet]. 2008;98(11):1762–8. Available from: http://www.pubmedcentral.nih.gov/articlerender.fcgi?artid=2410103&tool=pmcentrez&rendertype=abstract

6. Delea TE, Tappenden P, Sofrygin O, Browning D, Amonkar MM, Karnon J, et al. Cost-effectiveness of lapatinib plus capecitabine in women with HER2+ metastatic breast cancer who have received prior therapy with trastuzumab. Eur J Heal Econ. 2012;13(5):589–603.

7. Delea TE, Hawkes C, Amonkar MM, Lykopoulos K, Johnston SRD. Cost-effectiveness of lapatinib plus letrozole in post-menopausal women with hormone receptor- and HER2-positive metastatic breast cancer. Breast Care. 2013;8(6):429–37.

8. Dionne F, Mitton C, Rassekh R, Brooks B, Ross C, Hayden M, et al. Economic impact of a genetic test for cisplatin-induced ototoxicity. Pharmacogenomics J [Internet]. Nature Publishing Group; 2012;12(4):359–359. Available from: http://dx.doi.org/10.1038/tpj.2011.15

9. Djalalov S, Beca J, Hoch JS, Krahn M, Tsao M-S, Cutz J-C, et al. Cost effectiveness of EML4-ALK fusion testing and first-line crizotinib treatment for patients with advanced ALK-positive non-small-cell lung cancer. J Clin Oncol [Internet]. United States; 2014;32(10):1012–9. Available from: http://ovidsp.ovid.com/ovidweb.cgi?T=JS&PAGE=reference&D=med8&NEWS=N&AN=24567430

10. Essers BAB, Van Helvoort-Postulart D, Prins MH, Neumann M, Dirksen CD. Does the inclusion of a cost attribute result in different preferences for the surgical treatment of primary basal cell carcinoma?: A comparison of two discrete-choice experiments. Pharmacoeconomics. 2010;28(6):507–20.

11. Garrison LP, Veenstra DL. The economic value of innovative treatments over the product life cycle: The case of targeted trastuzumab therapy for breast cancer. Value Heal [Internet]. International Society for Pharmacoeconomics and Outcomes Research (ISPOR); 2009;12(8):1118–23. Available from: http://dx.doi.org/10.1111/j.1524-4733.2009.00572.x

12. Garrison LP, Lalla D, Brammer M, Babigumira JB, Wang B, Perez EA. Assessing the potential cost-effectiveness of retesting IHC0, IHC1+, or FISH-negative early stage breast cancer patients for HER2 status. Cancer. 2013;119(17):3113–22.

13. Hornberger J, Chien R, Krebs K, Hochheiser L. US insurance program’s experience with a multigene assay for early-stage breast cancer. Am J Manag Care. 2011;17(5).

14. Ito K, Elkin E, Blinder V, Keating N, Choudhry N. Cost-effectiveness of full coverage of aromatase inhibitors for Medicare beneficiaries with early breast cancer. Cancer. 2013;119(13):2494–502.

15. Klang SH, Hammerman A, Liebermann N, Efrat N, Doberne J, Hornberger J. Economic implications of 21-gene breast cancer risk assay from the perspective of an Israeli-managed health-care organization. Value Heal [Internet]. International Society for Pharmacoeconomics and Outcomes Research (ISPOR); 2010;13(4):381–7. Available from: http://dx.doi.org/10.1111/j.1524-4733.2010.00724.x

16. Kondo M, Hoshi SL, Ishiguro H, Yoshibayashi H, Toi M. Economic evaluation of 21-gene reverse transcriptase-polymerase chain reaction assay in lymph-node-negative, estrogen-receptor-positive, early-stage breast cancer in Japan. Breast Cancer Res Treat. 2008;112:175–87.

17. Lala A, Berger JS, Sharma G, Hochman JS, Scott Braithwaite R, Ladapo JA. Genetic testing in patients with acute coronary syndrome undergoing percutaneous coronary intervention: A cost-effectiveness analysis. J Thromb Haemost. 2013;11(1):81–91.

18. Lee H-J, Lee T-J, Yang B-M, Min J. Cost–effectiveness analysis of adjuvant hormonal treatments for women with postmenopausal hormone-receptor positive early breast cancer in the Korean context. J Breast Cancer. 2010;13(3):286–98.

19. Lidgren M, Wilking N, Jonsson B, Rehnberg C. Cost-effectiveness of HER2 testing and trastuzumab therapy for metastatic breast cancer. Acta Oncol (Madr). 2008;47(6):1018–28.

20. Lorenzana SB, Hughes MD, Grinsztejn B, Collier AC, Luz PM, Freedberg KA, et al. Genotype assays and third-line ART in resource-limited settings: A simulation and cost-effectiveness analysis of a planned clinical trial. Aids. 2012;26(9):1083–93.

21. Manchanda R, Legood R, Burnell M, McGuire A, Raikou M, Loggenberg K, et al. Cost-effectiveness of Population Screening for BRCA Mutations in Ashkenazi Jewish Women Compared With Family History–Based Testing. JNCI J Natl Cancer Inst [Internet]. 2015 Jan [cited 2017 Jul 26];107(1):380. Available from: http://www.ncbi.nlm.nih.gov/pubmed/25435542

22. McCowan C, Wang S, Thompson AM, Makubate B, Petrie DJ. The value of high adherence to tamoxifen in women with breast cancer: a community-based cohort study. Br J Cancer [Internet]. Nature Publishing Group; 2013;109(5):1172–80. Available from: http://www.ncbi.nlm.nih.gov/pmc/articles/PMC3778308/pdf/bjc2013464a.pdf

23. Medical Advisory Secretariat. Epidermal Growth Factor Receptor Mutation (EGFR) Testing for Prediction of Response to EGFR-Targeting Tyrosine Kinase Inhibitor (TKI) Drugs in Patients with Advanced Non-Small-Cell Lung Cancer: An Evidence-Based Analysis [Internet]. Ont Health Technol Assess Ser. 2010. Available from: http://www.ncbi.nlm.nih.gov/pubmed/23074402%5Cnhttp://www.ncbi.nlm.nih.gov/pmc/articles/PMC3377519/pdf/ohtas-10-48.pdf

24. Medical Advisory Secretariat. Gene Expression Profiling for Guiding Adjuvant Chemotherapy Decisions in Women with Early Breast Cancer: An Evidence-Based and Economic Analysis [Internet]. Ontario Health Technology Assessment Series. 2010. 1-57 p. Available from: http://www.ncbi.nlm.nih.gov/pubmed/15591335%5Cnhttp://www.ncbi.nlm.nih.gov/pmc/articles/PMC3382301/

25. Patrick AR, Avorn J, Choudhry NK. Cost-effectiveness of genotype-guided warfarin dosing for patients with atrial fibrillation. Circ Cardiovasc Qual Outcomes. 2009;2(5):429–36.

26. Retèl VP, Joore M a., Knauer M, Linn SC, Hauptmann M, Harten WH Van. Cost-effectiveness of the 70-gene signature versus St. Gallen guidelines and Adjuvant Online for early breast cancer. Eur J Cancer. 2010;46(8):1382–91.

27. Retèl VP, Joore M a, Linn SC, Rutgers EJT, van Harten WH. Scenario drafting to anticipate future developments in technology assessment. BMC Res Notes [Internet]. 2012;5:442. Available from: http://www.pubmedcentral.nih.gov/articlerender.fcgi?artid=3444406&tool=pmcentrez&rendertype=abstract

28. Romanus D, Cardarella S, Cutler D, Landrum MB, Lindeman NI, Gazelle GS. Cost-effectiveness of multiplexed predictive biomarker screening in non-small-cell lung cancer. J Thorac Oncol [Internet]. 2015;10(4):586–94. Available from: http://www.pubmedcentral.nih.gov/articlerender.fcgi?artid=4395466&tool=pmcentrez&rendertype=abstract

29. Rubinstein WS, Jiang H, Dellefave L, Rademaker AW. Cost-effectiveness of population-based BRCA1/2 testing and ovarian cancer prevention for Ashkenazi Jews: A call for dialogue. Genet Med [Internet]. 2009 Sep 14 [cited 2017 Jul 26];11(9):629–39. Available from: http://www.ncbi.nlm.nih.gov/pubmed/19606050

30. Saokaew S, Tassaneeyakul W, Maenthaisong R, Chaiyakunapruk N. Cost-effectiveness analysis of HLA-B*5801 testing in preventing allopurinol-induced SJS/TEN in Thai population. PLoS One. 2014;9(4):1–9.

31. Segui MA, Crespo C, Cortes J, Lluch A, Brosa M, Becerra V, et al. Genomic profile of breast cancer: cost-effectiveness analysis from the Spanish National Healthcare System perspective. Expert Rev Pharmacoecon Outcomes Res [Internet]. England; 2014;14(6):889–99. Available from: http://ovidsp.ovid.com/ovidweb.cgi?T=JS&PAGE=reference&D=med8&NEWS=N&AN=25213317

32. Thompson AJ, Newman WG, Elliott RA, Roberts SA, Tricker K, Payne K. The cost-effectiveness of a pharmacogenetic test: A trial-based evaluation of TPMT genotyping for azathioprine. Value Heal [Internet]. Elsevier; 2014;17(1):22–33. Available from: http://dx.doi.org/10.1016/j.jval.2013.10.007

33. Vanderlaan BF, Broder MS, Chang EY, Oratz R, Bentley TGK. Cost-effectiveness of 21-gene assay in node-positive, early-stage breast cancer. Am J Manag Care. 2011;17(7):455–64.
